# CCDC41 Drives Oocyte Meiotic Progression by Promoting Rab11a/Rab7‐Positive Vesicle Fusion with Target Membranes

**DOI:** 10.1002/advs.202504665

**Published:** 2025-12-02

**Authors:** Ying Tian, Jiatong Li, Jingyi Kang, Xiangning Xu, Bicheng Wang, Shuo Lou, Jingyu Li, Yuying Yang, Yanbing Zhang, Yangzi Zheng, Jing Weng, Yuanjing Liang, Wei Ma

**Affiliations:** ^1^ Department of Histology and Embryology School of Basic Medical Sciences Capital Medical University Beijing 100069 P. R. China; ^2^ Department of Occupational Therapy China Rehabilitation Research Center Beijing 100068 P. R. China; ^3^ Department of Science Education and Foreign Affairs Jinan Seventh People's Hospital Jinan 250100 P. R. China; ^4^ Department of Human Reproductive Medicine Beijing Obstetrics and Gynecology Hospital Capital Medical University Beijing 100026 P. R. China

**Keywords:** autophagy, CCDC41, CTSB, lysosome, mouse oocytes

## Abstract

Coiled‐coil domain‐containing protein 41 (CCDC41), a core component of centriolar distal appendages involved in centriole assembly and ciliary vesicle docking, has remained functionally uncharacterized in oocyte meiosis. Here, it is demonstrated that CCDC41 is a versatile player in three key steps of oocyte meiosis. First, CCDC41 depletion significantly impaired meiotic resumption, a defect mechanistically linked to reduced Cyclin B1 accumulation, compromised cyclin‐dependent kinase 1 (CDK1) activation, and dysregulation of the anaphase‐promoting complex/cyclosome (APC/C) co‐activators Cdc20 homolog 1 (CDH1) and cell division cycle 20 (CDC20). Notably, this arrest is rescued by pharmacological inhibition of CDC20. Second, CCDC41 knockdown disrupted spindle cortical migration, a defect ascribed to impaired fusion of RAS oncogene family (Rab11a)‐positive vesicles with the plasma membrane, which in turn prevented the anchoring of cytoplasmic F‐actin to the cortex. Third, CCDC41 depletion accelerated anaphase onset by prematurely silencing the spindle assembly checkpoint and enhancing CDC20‐mediated Cyclin B1 degradation. Mechanistically, CCDC41 localizes to lysosomes, and its loss delays RAS oncogene family member 7 (Rab7)‐positive late endosome fusion with these organelles, causing cytoplasmic dispersion of active cathepsin. Critically, pharmacological or genetic inhibition of cathepsin B restored meiotic progression in CCDC41‐deficient oocytes. Collectively, the findings establish that CCDC41 is essential for faithful meiotic completion, it regulates CDC20 activity through promoting cathepsin delivery to lysosomes, and ensures proper spindle migration via Rab11a‐dependent F‐actin anchoring.

## Graphical abstract

CCDC41 is essential for meiotic maturation in mouse oocytes through regulating Rab7‐positive endosomes fusion with lysosomes and Rab11a‐positive vesicle fusion with the plasma membrane.

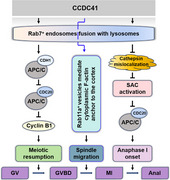

## 1 Introduction

Successful oocyte meiosis, which involves at least three critical stages, meiotic resumption, spindle migration, and anaphase onset, is essential for generating genetically competent gametes. Errors during this process can disrupt chromosome segregation and cytokinesis, thereby compromising the genetic integrity of the mature oocyte and the proper inheritance of maternal factors. Such defects ultimately impair fertilization capacity and subsequent developmental potential.^[^ ^1^ ^]^

Oocyte meiotic resumption is phenotypically marked by the breakdown of the germinal vesicle (GVBD) and is initiated by the complete activation of the maturation‐promoting factor (MPF), which involves the sustained accumulation of Cyclin B1 and the dephosphorylation of cyclin‐dependent kinase 1 (CDK1) at its inhibitory sites (T14 and Y15).^[^ ^2^ ^]^ Cyclin B1 is continuously targeted for degradation by the anaphase‐promoting complex/cyclosome (APC/C) in conjunction with its co‐activator, Cdc20 homolog 1 (CDH1) (APC/C^CDH1^) at meiotic prophase I, and only begins to rise as the concentration of CDH1 decreases.^[^ ^3^ ^]^ CDH1 stability during prophase I is regulated by benzimidazole 1‐related 1 (BubR1), a core component of the spindle assembly checkpoint (SAC). Loss of BubR1 induces premature CDH1 degradation and germinal vesicle breakdown (GVBD) in oocytes. ^[^ ^4^ ^]^ Interestingly, in multiple somatic cell lines, the stability of BubR1 and other BUB family proteins, including budding uninhibited by benzimidazoles 3 (BUB3) and budding uninhibited by benzimidazoles 1 (BUB1), is controlled by Sirtuin 1 (Sirt1).^[^ ^5^ ^]^ Sirt1 has recently been identified as a novel mammalian substrate of nuclear autophagy, with its protein abundance negatively correlated with lysosomal activity.^[^ ^6^, ^7^ ^]^ However, it is currently unknown whether lysosomal activity plays a role in modulating oocyte arrest at prophase I through the Sirt1‐BubR1‐CDH1 signaling pathway.

Following meiotic resumption, the spindle assembles progressively during prometaphase I and metaphase I (MetI). Upon formation, this spindle migrates to the cortex, a critical step for asymmetric division that is driven by the dynamics of the F‐actin network and its coupling to the plasma membrane.^[^ ^8^ ^]^ This process is initiated by the recruitment of actin‐nucleating factors, including formin 2 (FMN2) and the actin‐related protein 2/3 complex (Arp2/3 complex), to Rab11a (RAB11A, member RAS oncogene family)‐positive vesicles.^[^ ^9^, ^10^ ^]^ These vesicles undergo dynamic fusion and anchor to the oocyte cortex, thereby generating and anchoring the F‐actin network required for spindle translocation.^[^ ^10^ ^]^ We previously found that hyperactivated autophagic flux disrupts this vesicle fusion machinery, impairing spindle migration and asymmetric division, causing the formation of an enlarged first polar body (1^st^ PB) in oocytes at metaphase II (MetII), indicating that tightly regulated, low‐level autophagy activity is essential for successful spindle positioning in oocytes.^[^ ^11^ ^]^

The metaphase‐to‐anaphase transition in oocyte meiosis I is governed by the spindle assembly checkpoint (SAC) signaling. At unattached kinetochores, the SAC proteins BubR1, BUB3, and mitotic arrest deficient‐like 2 (MAD2) form the mitotic checkpoint complex (MCC) with cell division cycle 20 (CDC20), inhibiting APC/C^CDC20^ activation and thereby preventing anaphase onset.^[^ ^12^ ^]^ Once all chromosomes achieve bipolar attachment to the spindle and come under tension, SAC proteins dissociate from kinetochores. This releases CDC20, allowing it to activate the APC/C.^[^ ^13^ ^]^ Active APC/C^CDC20^ then triggers anaphase by targeting Securin and Cyclin B1 for ubiquitin‐mediated degradation.^[^ ^14^, ^15^ ^]^ While the precise, lysosomal release of cathepsin B (CTSB) is known to be essential for SAC activation and faithful chromosome segregation in mitosis,^[^ ^16^, ^17^, ^18^ ^]^ paradoxical evidence suggests that CTSB overactivation can disrupt the SAC in mouse oocytes.^[^ ^18^, ^19^ ^]^ Thus, the full scope of lysosomal functions in regulating the SAC and anaphase onset during meiosis remains an open question.

Coiled‐coil domain‐containing protein 41 (CCDC41), a constituent of the distal appendage proteins (DAPs) of the mother centrioles, partially colocalizes with the Golgi complex and collaborates with infraglabellar transport protein 20 (IFT20) to guide the docking of ciliary vesicles to the mother centriole and their subsequent fusion with the plasma membrane during the assembly of cilia in somatic cells.^[^ ^20^, ^21^ ^]^ Centrioles degenerate early in mammalian oogenesis, leaving the existence and function of centriole structural components, including CCDC41, unsolved in the context of oocyte meiotic progression. Here, we discovered that CCDC41 is expressed in mouse oocytes and is essential for faithful completion of meiotic progression. It achieves this by regulating two critical events: controlling RAS oncogene family member 7 (Rab7)‐positive endosome fusion with lysosomes to ensure proper meiotic resumption and anaphase onset, and mediating Rab11a‐positive vesicle anchoring to the plasma membrane to facilitate correct spindle migration.

## 2 Results

### 2.1 CCDC41 is Required for Meiosis Resumption in Mouse Oocytes

**Figure 1:**
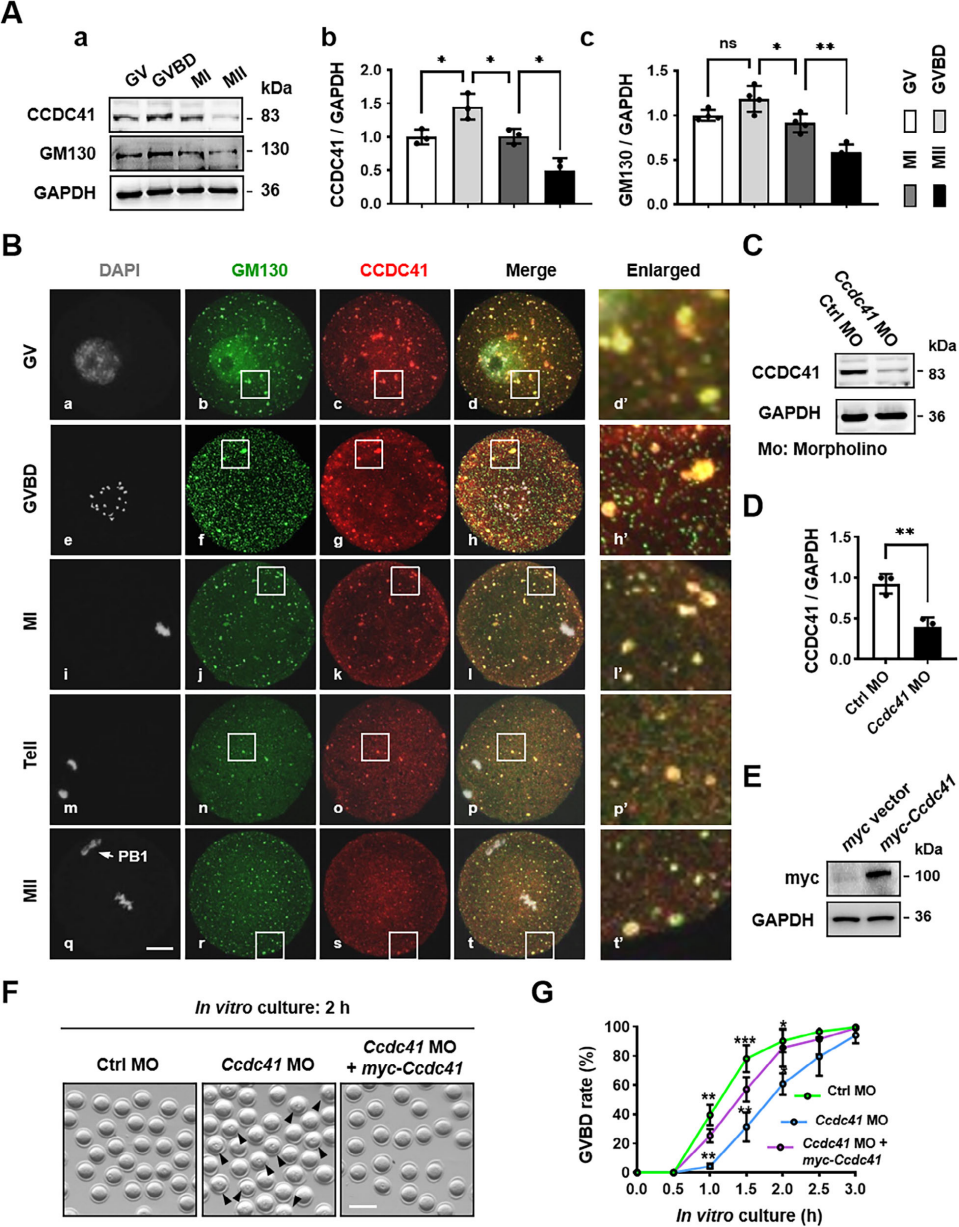
CCDC41 is required for meiosis resumption in mouse oocytes. A) Western blot and quantitative analysis of CCDC41 and GM130 protein expression in mouse oocytes at different meiotic stages. B) Immunofluorescence analysis of CCDC41 and GM130 subcellular localization representative images of CCDC41 (red) co‐localization with GM130 (green) in oocytes. Nuclear DNA is stained with DAPI and displayed in gray. The area within the white frame is enlarged (5 × magnification) to provide a detailed view. Scale bar = 20 µm. C) Western blot analysis of CCDC41 expression in oocytes injected with *Ccdc41*‐specific morpholino oligomers (MO) or control morpholino (Ctrl MO). Each sample consisted of 90 oocytes. D) Quantitative assessment of CCDC41 protein levels across different experimental groups of oocytes. E) Western blot analysis confirmed efficient expression of myc‐CCDC41 in mouse oocytes, with each sample comprising 60 oocytes. F) Representative microscopy images of oocytes treated with Ctrl MO, *Ccdc41* MO, and *Ccdc41* MO + *myc‐Ccdc41* complementary RNA (cRNA) following a 2‐h maturation culture. Arrows indicate oocytes at the GV stage. G) Statistical analysis of GVBD rates at consecutive time points during in vitro culture across the Ctrl MO (n = 113), *Ccdc41* MO (n = 118), and *Ccdc41* MO + *myc‐Ccdc41* cRNA (n = 99) groups. The experiment was repeated three times independently. P values were determined using one‐way ANOVA for A, G, and unpaired Student's t‐tests (two‐tailed) for D. Statistical significance is denoted as ^*^P < 0.05, ^**^P < 0.01, ^***^P < 0.001.

Western blot analysis demonstrated dynamic expression of CCDC41 in mouse oocytes, which increased from the germinal vesicle (GV) stage, peaked at GVBD, and subsequently decreased by MetII. This pattern paralleled that of the cis‐Golgi matrix protein GM130 in oocytes (**Figure** **1**A). Immunofluorescence demonstrated that CCDC41 exhibited a multipoint distribution within oocytes during meiotic maturation, predominantly as multiple foci of diverse sizes distributed across the cytoplasm. CCDC41 foci were colocalized with GM130, a key vesicle marker, from GV to MetII stage (Figure 1B), but surprisingly, absent at the microtubule organizing centers (MTOCs), as identified by Pericentrin (Figure S1A, Supporting Information). Western blot confirmed efficient translation of the microinjected cRNA for *eGFP‐Ccdc41* in oocytes (Figure S1B‐i, Supporting Information). Live‐cell imaging further showed that the eGFP signal appeared as dot‐like clusters distributed across the cytoplasm, predominantly aggregated in the region of the putative spindle apparatus at MetI stage. A subset of these clusters was also found concentrated within the nucleus at GV stage (Figure S1B‐ii, Supporting Information). The unique distribution pattern of CCDC41 suggests its possible involvement in meiosis resumption and spindle migration in oocytes.

Immunoblot analysis confirmed a marked reduction in CCDC41 protein levels in oocytes injected with *Ccdc41*‐targeting oligonucleotides (MO) (Figure 1C,D). Furthermore, in vitro‐transcribed *myc‐Ccdc41* cRNA was successfully translated in oocytes (Figure 1E). In comparison to control oocytes, oocytes injected with *Ccdc41* MO exhibited a significant delay in GVBD at 1, 1.5, 2, and 2.5 h of in vitro maturation culture (Figure 1F,G). This delay was markedly rescued at each time point by co‐injection of *myc‐Ccdc41* cRNA. Interestingly, at 3 h of culture, no significant difference in GVBD rates was observed among the Control (Ctrl) MO, *Ccdc41* MO, and *Ccdc41* MO + *myc‐Ccdc41* cRNA groups (Figure 1G).

### 2.2 CCDC41 Regulates Meiotic Resumption Through Modulating the Levels of CDH1 and CDC20

**Figure 2:**
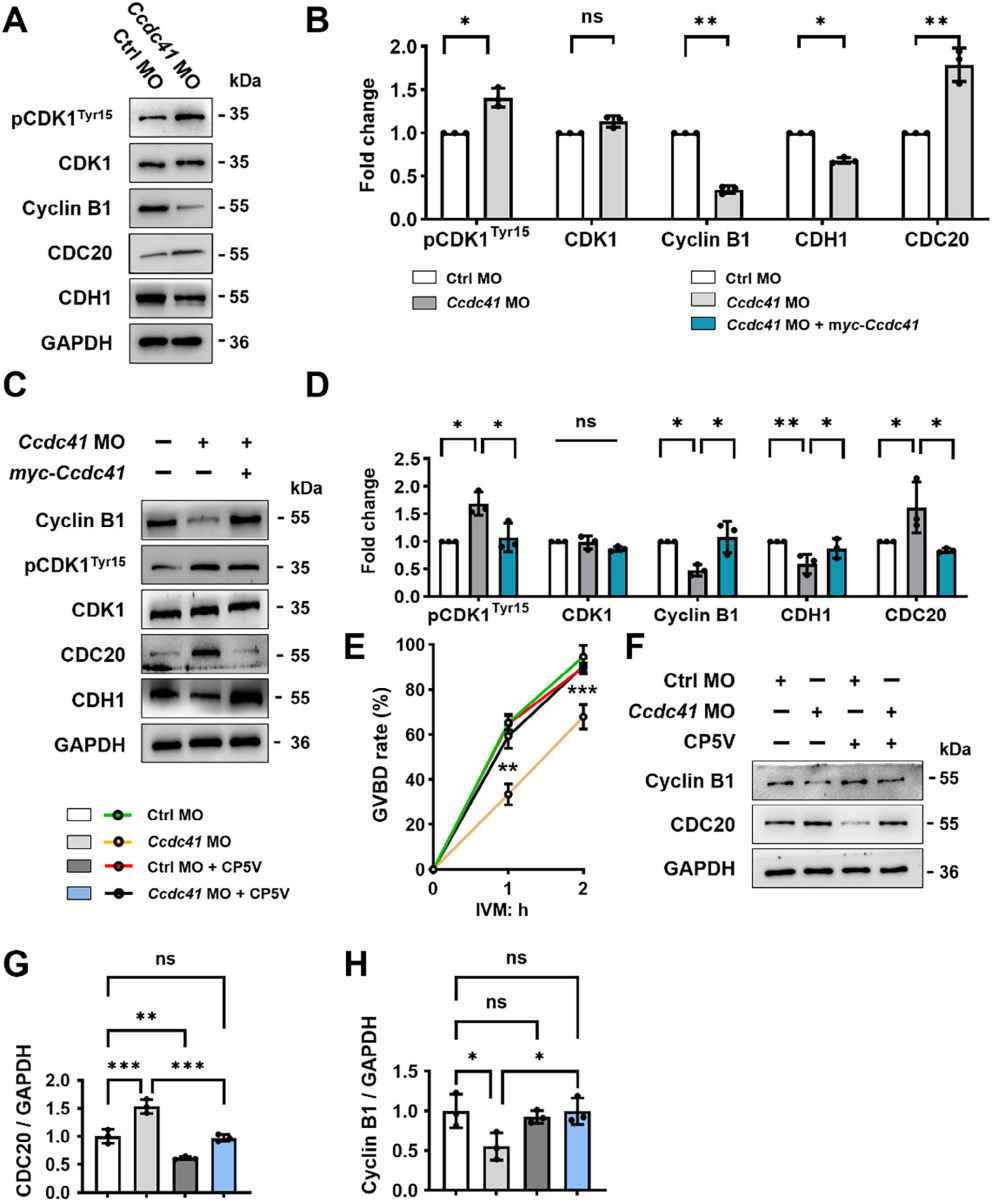
CCDC41 loss delayed oocyte meiotic resumption by elevating CDC20 level. A) Western blot analysis showing protein levels in GV oocytes from the Ctrl and *Ccdc41* MO groups. B) Quantitative analysis of protein levels in Ctrl and *Ccdc41* MO‐treated GV oocytes. C) Western blot images illustrating protein level alterations in GV oocytes treated with Ctrl MO, *Ccdc41*MO, and *Ccdc41* MO + *myc‐Ccdc41* cRNA. D) Quantitative analysis of protein levels in Ctrl MO, *Ccdc41* MO, and *Ccdc41*MO + *myc‐Ccdc41* cRNA‐treated GV oocytes, respectively. E) Quantitative analysis of GVBD rate in oocytes cultured for 1 and 2 h from the Ctrl and *Ccdc41* MO groups, with or without prior treatment with CP5V (the specific CDC20 inhibitor). F) Western blot analysis of Cyclin B1 and CDC20 expression in the various treatment groups. G,H) Quantitative assessment of Cyclin B1 and CDC20 protein levels in GV oocytes across different treatments. Each sample comprised 60–150 oocytes. The experiments were repeated at least three times independently. P‐values were calculated using unpaired Student's t‐tests (two‐tailed) for B and one‐way ANOVA for D–E, G–H. Statistical significance is denoted as ^*^P < 0.05, ^**^P < 0.01, ^***^P < 0.001.

In mouse oocytes, meiotic resumption failure is associated with impaired Cdk1 activity binding to its regulatory subunit Cyclin B1. Changes in the levels of Cyclin B1 and/or the regulatory protein CDH1 of its E3 ubiquitin ligase APC/C^CDH1^ could inhibit entry into M phase. We observed ≈50% increase in the levels of phosphorylated CDK1 (p‐CDK1) in oocytes depleted of CCDC41, which was effectively reversed in CCDC41‐rescued oocytes. Conversely, the levels of Cyclin B1 were markedly reduced in CCDC41‐deficient oocytes but significantly recovered upon co‐administration of *myc‐Ccdc41* cRNA. In parallel with the alterations in CDK1 and Cyclin B1, the levels of CDH1 were significantly decreased, while those of CDC20 were notably increased in CCDC41‐depleted oocytes. This pattern of change was reversed by the introduction of exogenous *Ccdc41* cRNA (**Figure** **2**A–D). These findings suggest that CCDC41 may facilitate the activation of MPF by modulating CDC20‐mediated Cyclin B1 degradation during oocyte meiotic resumption.

Consistently, treatment with the specific CDC20 inhibitor, CP5V,^[^ ^22^ ^]^ effectively alleviated the phenotypic defects associated with CCDC41 loss in GV oocytes. Specifically, the percentage of GVBD in the *Ccdc41* MO group was restored to a level comparable to that of the control group following CP5V treatment (Figure 2E). This was consistent with the observed decrease in CDC20 and the increase in Cyclin B1 levels, as confirmed by western blot analysis (Figure 2F–H). These results reveal a striking contrast with previous studies, indicating that CCDC41 promotes meiotic resumption by counteracting CDC20 activity during APC/C^CDH1^‐dominated prometaphase I in oocytes.

### 2.3 CCDC41 is Required for Spindle Assembly and Migration in Oocytes

**Figure 3:**
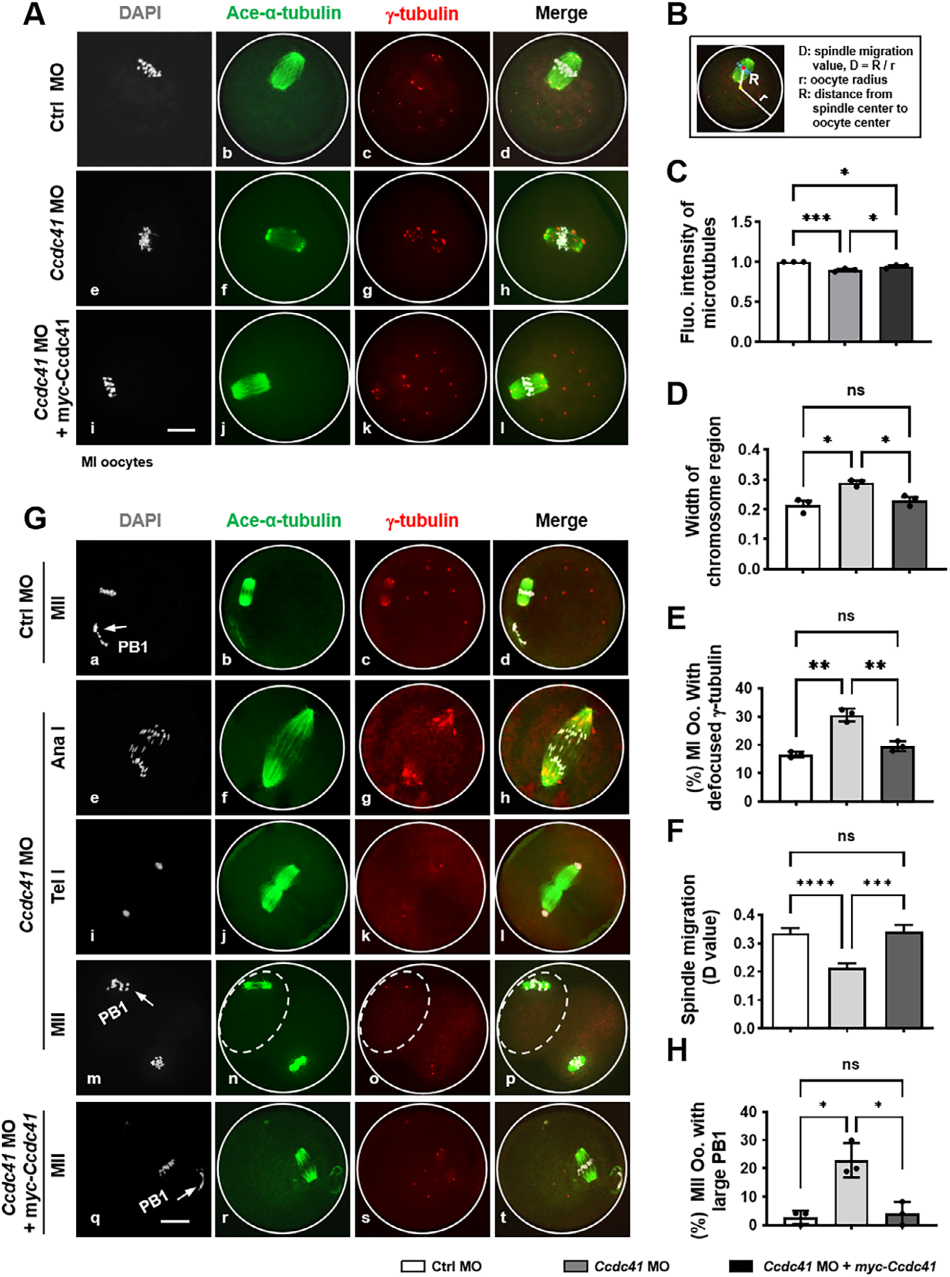
CCDC41 depletion disrupts spindle assembly, migration, and asymmetric division in oocytes. A) Microscopy images of MetI oocytes from the Ctrl MO, *Ccdc41* MO, and *Ccdc41* MO + *myc‐Ccdc41* cRNA treatment groups. Scale bar = 20 µm. B) Schematic representation of the spindle migration calculation model. C‐F) Statistical evaluation of spindle migration distance (D value), fluorescence intensity of microtubules, width of chromosome region, and the percentage of oocytes with defocused polar MTOCs in the Ctrl MO (n = 96), *Ccdc41* MO (n = 98), and *Ccdc41* MO + *myc‐Ccdc41* cRNA (n = 87) groups. G) Representative microscopy images of oocytes at anaphase I (AnaI), telophase I (TelI), and MetII stages in the Ctrl MO, *Ccdc41* MO, and *Ccdc41* MO + *myc‐Ccdc41* cRNA groups. H) Quantification of the frequency of MetII oocytes with an enlarged first polar body (PB1) across the different treatment groups. Acetylated α‐tubulin (Ace‐α‐tubulin) was visualized in green, γ‐tubulin in red, and DNA in gray. Scale bar = 20 µm. The experiments were replicated at least three times independently. P‐values were determined using one‐way ANOVA. Statistical significance is indicated as ^*^P < 0.05, ^**^P < 0.01, ^***^P < 0.001, and ^****^P < 0.0001.

As demonstrated by immunofluorescence, the depletion of CCDC41 significantly impaired spindle architecture in MetI oocytes, featuring a decrease in microtubule density and the presence of un‐clustered γ‐tubulin‐containing MTOCs in fixed mouse oocytes at MetI stage, as visualized by immunostaining (**Figure** **3**A). Paralleling this structural defect, chromosomes failed to align at the metaphase plate and were instead observed in a scattered and disorganized configuration (Figure 3A). Quantitatively, the proportion of MetI oocytes with aberrant spindles and misaligned chromosomes was significantly higher in the *Ccdc41* MO group than in the control (Figure 3C–E). Obviously, these defects were significantly rescued upon expression of exogenous CCDC41 (Figure 3B–E). Furthermore, the spindle was mispositioned, failing to migrate to the cortex area and instead remaining centralized in MetI oocytes lacking CCDC41 (Figure 3A,F), causing the formation of enlarged PB1 at the MetII stage (Figure 3G). To quantify changes in spindle migration following MO treatment, we measured the oocyte spherical radius (R) and the distance (r) between the spindle center and the oocyte spherical center in MetI oocytes after immunofluorescence staining. The ratio of “R” to “r” (D) was then calculated to represent the relative spindle migration distance (Figure 3B). Statistical analysis revealed that the D value was significantly lower in the *Ccdc41* MO group compared to the control group, but this decrease was reversed by co‐injection of *myc‐Ccdc41* cRNA, with no significant difference in D value between the Ctrl MO and *Ccdc41* MO + *myc‐Ccdc41* cRNA groups (Figure 3F). These results clearly demonstrate that CCDC41 depletion delays spindle migration, accounting for the formation of larger PB1 in MetII oocytes (Figure 3G,H). Collectively, these findings indicate that CCDC41 is crucial for oocyte spindle organization and proper cortical positioning.

### 2.4 CCDC41 Regulates Spindle Migration via Promoting Rab11a‐Positive Vesicle‐Mediated F‐Actin Cortical Anchoring

Immunofluorescence demonstrated the disappearance of GM130 puncta in oocytes treated with the vesicle assembly inhibitor Brefeldin A (BFA) for 7 h, indicative of vesicle disassembly. This was accompanied by the loss of CCDC41 dot‐like signals. Following BFA removal and an 18‐min recovery period in fresh medium, both GM130 and CCDC41 reappeared as dot‐like aggregates in the oocytes, maintaining co‐localization (Figure S2A–C, Supporting Information). The duolink proximity ligation assay (PLA) revealed dense punctate signals in positive control oocytes incubated with antibodies against GM130 and Rab11a, contrasted with minimal signals in negative controls incubated with GM130 antibody alone. Equivalent bright PLA signals were observed in oocytes treated with antibodies against GM130 and CCDC41 or Rab11a and CCDC41. However, these antibody pairs detected scarce PLA signals in CCDC41‐depleted oocytes (Figure S2D,E, Supporting Information). This indicates close proximity between CCDC41 and vesicular proteins in mouse oocytes. Additionally, the depletion of CCDC41 via MO did not impact the absolute protein expression levels of GM130 and Rab11a (Figure S2F,G, Supporting Information). In line with PLA data, immunoblotting confirmed the presence of Rab11a and GM130 in oocyte lysates immunoprecipitated with CCDC41 antibody, but not with control IgG (Figure S2H, Supporting Information). These findings suggest that CCDC41 is associated with Rab11a‐positive vesicles involved in spindle migration during oocyte meiosis.

**Figure 4:**
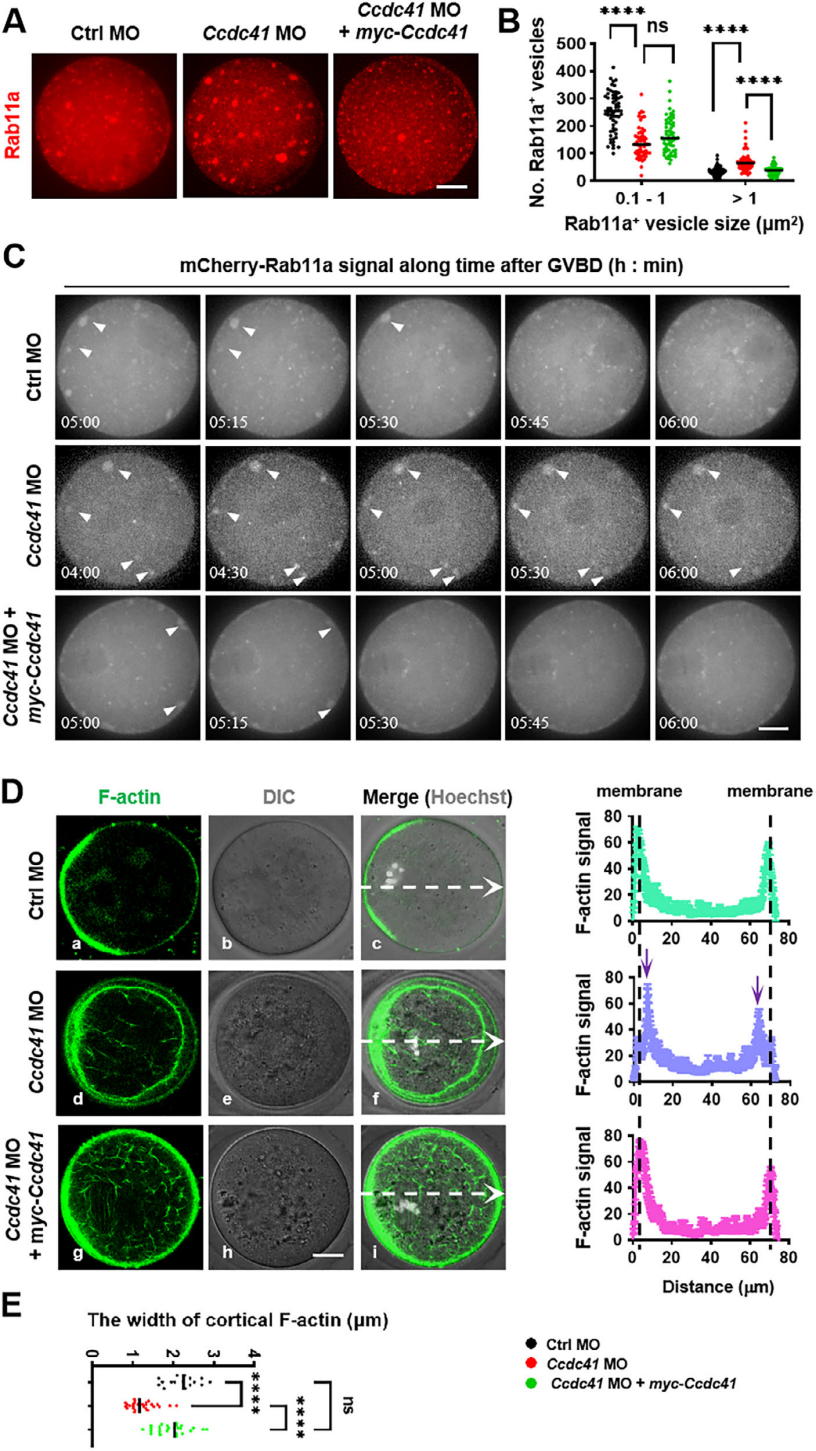
CCDC41 regulates spindle migration via promoting Rab11a‐positive vesicle‐mediated F‐actin cortical anchoring. A) Representative images of MetI oocytes from the Ctrl MO, *Ccdc41* MO, and *Ccdc4*1 MO + *myc‐Ccdc41* cRNA groups. Rab11a was stained in red. Scale bar = 20 µm. B) Statistical analysis of the number of large Rab11a‐positive vesicles (>1 µm^2^) and small Rab11a‐positive vesicles (0.1–1 µm^2^) in Ctrl MO (n = 60), *Ccdc41* MO (n = 57), and *Ccdc41* MO + *myc‐Ccdc41* cRNA (n = 60) groups. C) Live‐cell imaging of mCherry‐Rab11a in oocytes from Ctrl MO, *Ccdc41* MO, and *Ccdc41* MO + *myc‐Ccdc41* cRNA groups. See Supplementary Video for additional details. Maximal Z projections across the equatorial region of each oocyte. Scale bar = 20 µm. D) Representative images of F‐actin distribution after *Ccdc41* MO and/or *myc‐Ccdc41* cRNA expression in MetI oocytes. The curve represents the average fluorescence intensity profile of F‐actin along the line. The black dashed lines indicate the oocyte membrane, and violet arrows indicate peaks in actin intensity beneath the cell membrane. Maximal Z projections across the equatorial region of each oocyte. Scale bar = 20 µm. E) Statistical analysis of the width of cortical F‐actin in Ctrl MO (n = 16), *Ccdc41* MO (n = 28), and *Ccdc41* MO + *myc‐Ccdc41* cRNA (n = 27) groups. The experiment was performed in triplicate. P‐values were calculated using one‐way ANOVA. Statistical significance is denoted as ^****^P < 0.0001.

As expected, CCDC41 depletion disrupted Rab11a‐positive vesicle fusion dynamics, resulting in the formation of large vesicles in mouse oocytes. Immunofluorescence showed a significant increase in the number of large Rab11a‐positive vesicles (>1 µm^2^) and a decrease in small ones (<1 µm^2^) in oocytes from the *Ccdc41* MO group compared to the Ctrl MO group. This pattern was significantly reversed by the expression of *Ccdc41* cRNA (**Figure** **4**A,B). Additionally, the number of large GM130‐positive vesicles (>1 µm^2^) was also pronouncedly increased in oocytes treated with *Ccdc41* MO (Figure S2I,J, Supporting Information). mCherry‐tagged Rab11a was successfully expressed in GV oocytes and effectively traced vesicle dynamics throughout meiotic progression, as evidenced by time‐lapse imaging. In control oocytes, vesicles could be transported outward and fused with the plasma membrane within 15–30 min after GVBD, which is thought to promote the coupling of the F‐actin network to the cortical region, driving spindle migration once it is properly assembled. In contrast, in *Ccdc41* MO oocytes, vesicles failed to fuse with the plasma membrane up to 6 h after GVBD, although they could be transported outward and located immediately beneath the plasma membrane. The expression of exogenous CCDC41 restored vesicle fusion with the plasma membrane and cortical spindle migration in *Ccdc41* MO‐injected oocytes (Figure 4C; Video S1, Supporting Information).

To investigate which domain of CCDC41 is responsible for its interactions with GM130 and Rab11a, we generated CCDC41 deletion mutants in frame with myc and expressed them in NIH‐3T3 (Figures S3A, Supporting Information). The SMC domain (amino acids 39–622) of CCDC41 was essential for its interaction with both GM130 and Rab11a. Further mapping showed that the N‐terminal region (1–300 a.a.) was sufficient to mediate these interactions, whereas the C‐terminal fragment (301–691 a.a.) failed to bind either protein (Figure S3B, Supporting Information).

Confocal imaging using the fluorescence probe CellMask Green Actin Tracking Stain visualized a bright circular F‐actin ring along the cytoplasmic membrane in each control MetI oocyte, with only a few F‐actin bundles around the chromosome arrays, which were labeled with Hoechst 33342, and a dense actin signal in the local cell membrane near the chromosomes (Figure 4D a–c,E). This single F‐actin circle indicates efficient coupling of the cytoplasmic F‐actin network to the cell membrane, corresponding to efficient spindle migration. In contrast, MetI oocytes from the *Ccdc41* MO group displayed two F‐actin circles: an outer thin and narrow circle, which was totally overlapped with the cell membrane and likely represented cortical F‐actin, and an inner thicker circle that was significantly away from the cell membrane. Additionally, there were some F‐actin bundles distributed throughout the cytoplasm (Figure 4D d–f,E). The dual‐ringed assembly of F‐actin suggests that the cytoplasmic F‐actin meshwork was not coupled to the cell membrane, potentially leading to a loss of force driving spindle migration, as indicated by delayed spindle migration. Injection of *myc‐Ccdc41* cRNA effectively reversed the aberrant F‐actin assembly in oocytes treated with *Ccdc41* MO, resembling the control oocytes (Figure 4D g–i,E). Collectively, these findings suggest that CCDC41 modulates vesicle fusion with the plasma membrane, thereby ensuring the cortical anchoring of F‐actin and spindle migration toward the cortical region.

### 2.5 CCDC41 Ensures Timely Anaphase Onset by Stabilizing SAC Components

**Figure 5:**
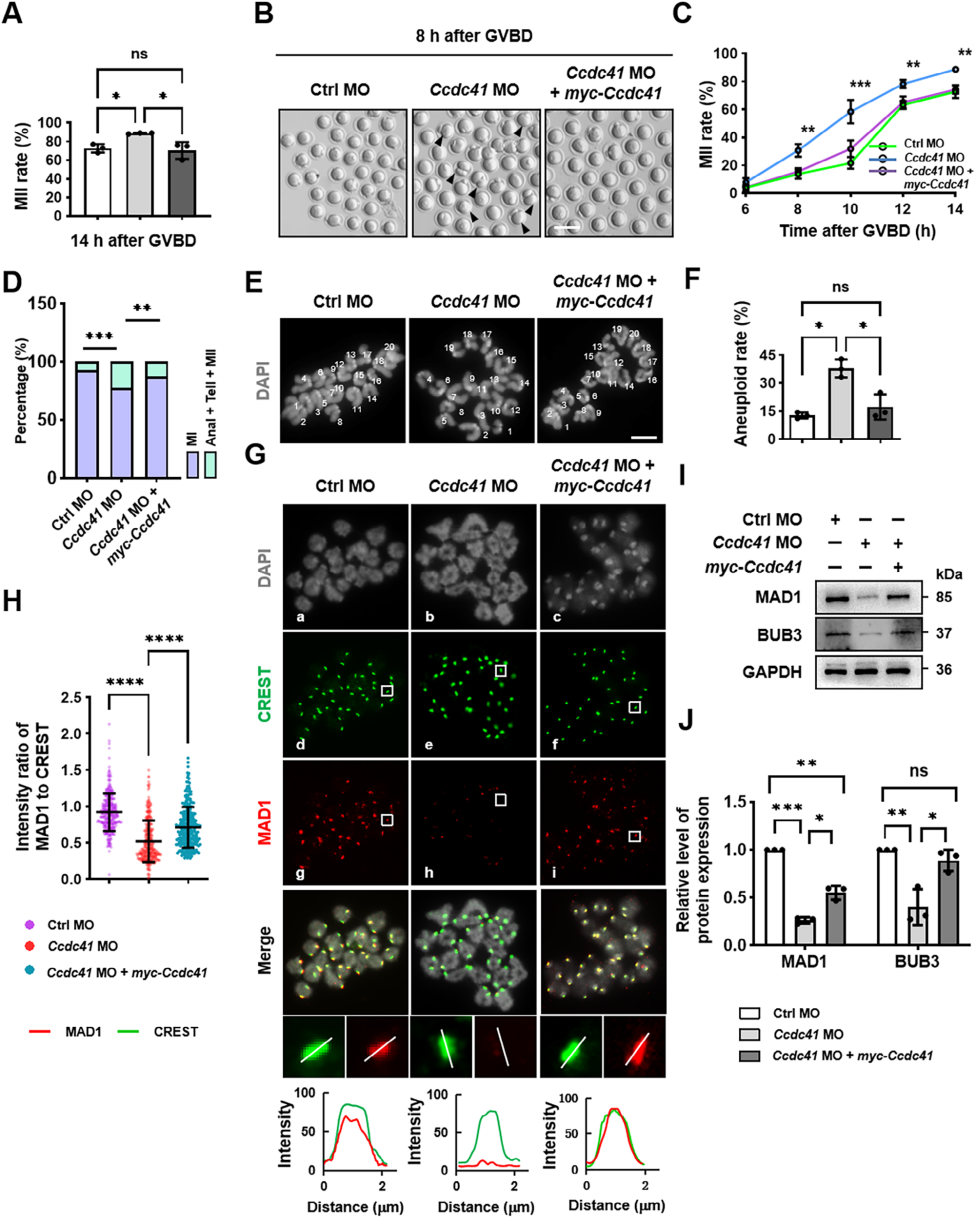
CCDC41 depletion results in spindle assembly checkpoint (SAC) dysfunction and aneuploidy in mouse oocytes. A) Statistical evaluation of MetII rate in Ctrl MO (n = 94), *Ccdc41* MO (n = 114), and *Ccdc41* MO + *myc‐Ccdc41* cRNA (n = 97) groups at 14 h post‐GVBD. B) Representative microscopy images of oocytes cultured for an additional 8 h after GVBD across the different treatment groups. Arrows denote oocytes with a prominently extruded, enlarged PB1. Scale bar = 200 µm. C) Statistical analysis of PB1 extrusion rates at consecutive time points following GVBD among the Ctrl MO (n = 94), *Ccdc41* MO (n = 114), and *Ccdc41* MO + *myc‐Ccdc41* cRNA (n = 97) groups. D) The proportion of oocytes overcoming MetI arrest induced by nocodazole treatment. E) Representative images of euploid and aneuploid MetII eggs. Scale bar = 2.5 µm. F) The frequency of aneuploid eggs was recorded for groups of Ctrl MO (n = 24), *Ccdc41* MO (n = 22), and *Ccdc41* MO + *myc‐Ccdc41* cRNA (n = 21). G) Oocytes were immunostained for MAD1, CREST, and DNA at 6 h post‐GVBD. White lines indicate the direction for measuring fluorescence intensity. Line graphs depict the fluorescence intensities of CREST (green line) and MAD1 (red line). Distances are measured in micrometers (µm). Scale bar = 2.5 µm. H) The relative fluorescence intensity ratio of MAD1 to CREST was quantified in oocytes from groups of Ctrl MO (n = 305), *Ccdc41* MO (n = 298), and *Ccdc41* MO + *myc‐Ccdc41* cRNA (n = 291). I) Protein levels of MAD1 and BUB3 were evaluated by western blot across the different treatment groups 4 h post‐GVBD. Each sample contained 120 oocytes. J) Quantitative assessment of MAD1 and BUB3 protein levels in oocytes across different treatments. The experiment was replicated three times independently. P‐values were calculated using one‐way ANOVA. Statistical significance is indicated as ^*^P < 0.05, ^**^P < 0.01, ^***^P < 0.001, ^****^P < 0.0001.

Following GVBD, oocytes were further cultured for 8, 10, 12, and 14 h, respectively, after which the rate of reaching MetII was significantly higher in the *Ccdc41* MO group compared to the Ctrl MO group. Quantitatively, the extrusion of PB1 occurred ≈2 h earlier in CCDC41‐depleted oocytes, an effect that was reversed by the co‐injection of exogenous *Ccdc41* cRNA (**Figure** **5**A–C). Furthermore, oocytes treated with *Ccdc41* MO, unlike those from the Ctrl MO and *Ccdc41* MO + *myc‐Ccdc41* cRNA groups, were able to overcome the MetI arrest induced by 500 nm nocodazole, a concentration that acutely disrupts spindle microtubule attachment to chromosomes without comprising spindle structure (Figure 5D). The chromosome counting revealed a significantly higher proportion of MetII oocytes with an abnormal number of chromosomes (more or less than 20) in the *Ccdc41* MO group compared to the Ctrl MO group. This trend was markedly reduced in the *Ccdc41* MO + *myc‐Ccdc41* cRNA group, which showed no significant difference from the Ctrl MO group (Figure 5E,F). Collectively, these findings demonstrate that CCDC41 loss results in premature anaphase onset, even in the presence of impaired microtubule‐chromosome attachments and subsequent chromosome missegregation, highlighting its pivotal role in the regulation of SAC signaling.

Immunofluorescence analysis revealed that CCDC41 localized to the centromeric region of chromosomes in MetI oocytes (Figure S4A, Supporting Information). Additionally, the SAC protein MAD1 (mitotic arrest deficient 1) displayed robust kinetochore labeling during prometaphase I in both control and CCDC41‐rescued oocytes, whereas its signal intensity was reduced by ≈50% in CCDC41‐depleted oocytes (Figure 5G,H). Western blot analysis revealed that CCDC41 depletion significantly reduced the protein levels of BuB3 and MAD1. Conversely, exogenous *Ccdc41* cRNA supplementation restored these levels, although they remained lower than in the control group (Figure 5I,J). Consistent with the changes in SAC activity, Cyclin B1 levels were significantly reduced in MetI oocytes from the *Ccdc41* MO group, whereas CDC20 and APC2 (anaphase‐promoting complex subunit 2) levels were elevated compared to the Ctrl MO group (Figure S4B,C, Supporting Information). These changes were reversed by co‐administration of *myc‐Ccdc41* cRNA. Furthermore, total ubiquitin (UB) levels were decreased in CCDC41‐depleted oocytes, an effect rescued by *Ccdc41* cRNA (Figure S4D,E, Supporting Information). The elevated CDC20 and APC2 levels likely enhance APC/C complex activity,^[^ ^23^ ^]^ thereby accelerating the ubiquitination‐mediated degradation of Cyclin B1 and triggering anaphase onset. The proteasome inhibitor MG132^[^ ^24^ ^]^ was able to partially rescue the elevated rate of the defective spindle and entry into MetII in CCDC41‐depleted oocytes (Figure S4F–H, Supporting Information) but did not correct spindle migration and large PB1 formation (Figure S4I,J, Supporting Information). These results suggest that CCDC41 knockdown induces premature SAC silencing and APC/C^CDC20^ activation, leading to premature degradation of Cyclin B1, anaphase onset, and chromosome segregation errors.

### 2.6 Abnormal Lysosomal Dynamics in CCDC41‐Depleted Oocytes

Western blot analysis revealed increased SQSTM1 accumulation in *Ccdc41* MO‐treated oocytes, while the levels of autophagy protein 5 (ATG5) and microtubule‐associated protein 1 light chain 3‐II (LC3‐II) remained unchanged (Figure S5A,B, Supporting Information). Co‐injection of *myc‐Ccdc41* cRNA restored SQSTM1 to control levels without altering ATG5 or LC3‐II (Figure S5C–E, Supporting Information). Although immunofluorescence analysis indicated no difference in LC3 puncta number between groups (Figure S5F,G, Supporting Information), suggesting intact autophagosome formation, transmission electron microscopy (TEM) revealed enlarged autolysosomes and an increased number of smaller lipid droplets (LDs) in *Ccdc41* MO‐treated oocytes (Figure S6A,B, Supporting Information). Notably, pharmacological inhibition of autophagy with Bafilomycin A1 (Baf A1) significantly enhanced the tightly colocalized signals of the lysosomal marker Lysotracker and the lipid probe LD540 (Figure S6C,D, Supporting Information). These findings collectively indicate that CCDC41 depletion impairs the lysosomal degradation of LDs, a hallmark of defective autophagy. These defects were rescued by *myc‐Ccdc41* cRNA (Figure S6A,B, Supporting Information). Together, these findings indicate that autophagosome‐lysosome fusion proceeds normally in CCDC41‐depleted oocytes, but the resulting autolysosomes are dysfunctional.

**Figure 6:**
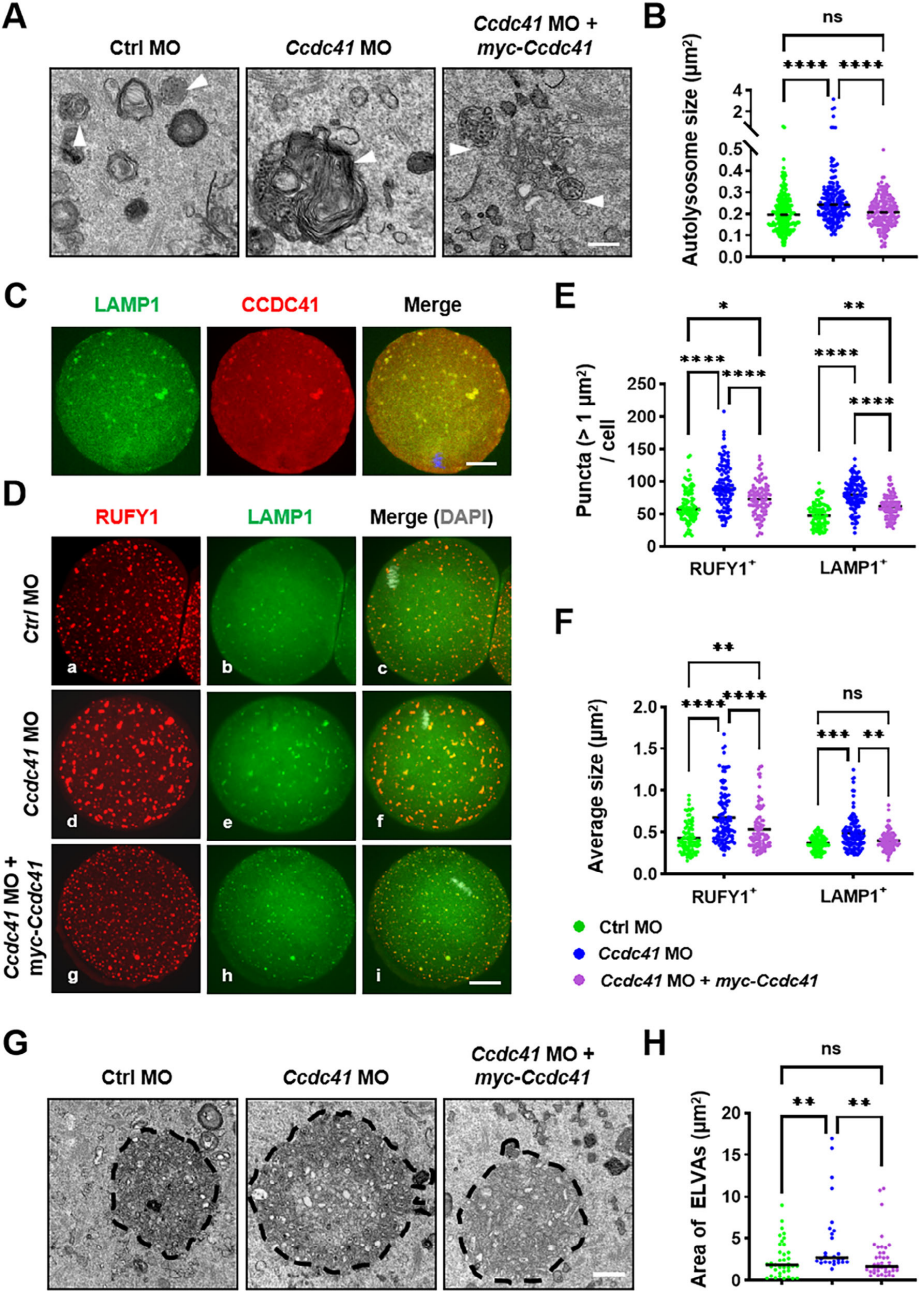
CCDC41 depletion causes lysosome over‐aggregation. A) Representative electron micrographs of MetI oocytes from the Ctrl MO, *Ccdc41* MO, and *Ccdc41* MO + *myc‐Ccdc41* cRNA groups, with arrowheads indicating autolysosomes. Scale bar = 500 nm. B) Quantification of autolysosome area in Ctrl MO (n = 235), *Ccdc41* MO (n = 124), and *Ccdc41* MO + *myc‐Ccdc41* cRNA (n = 202) groups. C) Immunofluorescence image of MetI oocytes immunolabeled with anti‐CCDC41 and anti‐LAMP1 antibodies. Scale bar = 20 µm. D) Representative images of oocytes immunolabeled with anti‐RUFY1 and anti‐LAMP1 in Ctrl MO, *Ccdc41* MO, and *Ccdc41* MO + *myc‐Ccdc41* cRNA groups 6 h after GVBD. Scale bar = 20 µm. E‐F) Quantification of the number and area of LAMP1 and RUFY1 (>1 µm^2^) in Ctrl MO (n = 87), *Ccdc41* MO (n = 110), and *Ccdc41* MO + *myc‐Ccdc41* + cRNA (n = 93) oocytes. G) Representative electron micrographs of early endosome‐like vesicles (ELVAs) in MetI oocytes from Ctrl MO, *Ccdc41* MO, and *Ccdc41* MO + *myc‐Ccdc41* cRNA groups. Scale bar = 500 nm. H) Quantification of the area of ELVAs (>1 µm^2^) in Ctrl MO (n = 37), *Ccdc41* MO (n = 26), and *Ccdc41* MO + *myc‐Ccdc41* cRNA (n = 40) groups. The experiment was performed three times independently. P‐values were calculated using one‐way ANOVA. Statistical significance is indicated as ^**^P < 0.01, ^***^P < 0.001, and ^****^P < 0.0001.

Immunofluorescence imaging revealed that CCDC41 colocalizes with lysosome‐associated membrane protein 1 (LAMP1) in mouse oocytes (**Figure** **6**C). In MetI oocytes, *Ccdc41* depletion significantly reduced the total number of LAMP1‐positive lysosomes. Conversely, the average area of these lysosomes was increased, with a significant rise in the number of large lysosomes (>1 µm^2^). These lysosomal defects were successfully rescued by the introduction of exogenous *Ccdc41* cRNA (Figure 6Db,e,h,E,F). Taken together with the preceding data, these findings indicate that CCDC41 loss impairs lysosomal biogenesis.^[^ ^25^ ^]^

In oocytes, endolysosomal vesicular assemblies (ELVAs) contain actin‐nucleating vesicles and depend on Rab11a for cortical movement.^[^ ^19^ ^]^ RUFY1, a recycling endosome regulator,^[^ ^26^, ^27^, ^28^ ^]^ organizes the ELVA structure.^[^ ^19^ ^]^ Immunofluorescence showed RUFY1 colocalization with LAMP1. *Ccdc41* MO treatment increased large RUFY1‐positive vesicles (>1 µm^2^), effects reversed by *myc‐Ccdc41* cRNA (Figure 6D,F). TEM further revealed fewer but larger ELVAs in CCDC41‐depleted oocytes, also rescued by cRNA (Figure 6D,F). Together, these data indicate that CCDC41 depletion impairs ELVA dynamics.

**Figure 7:**
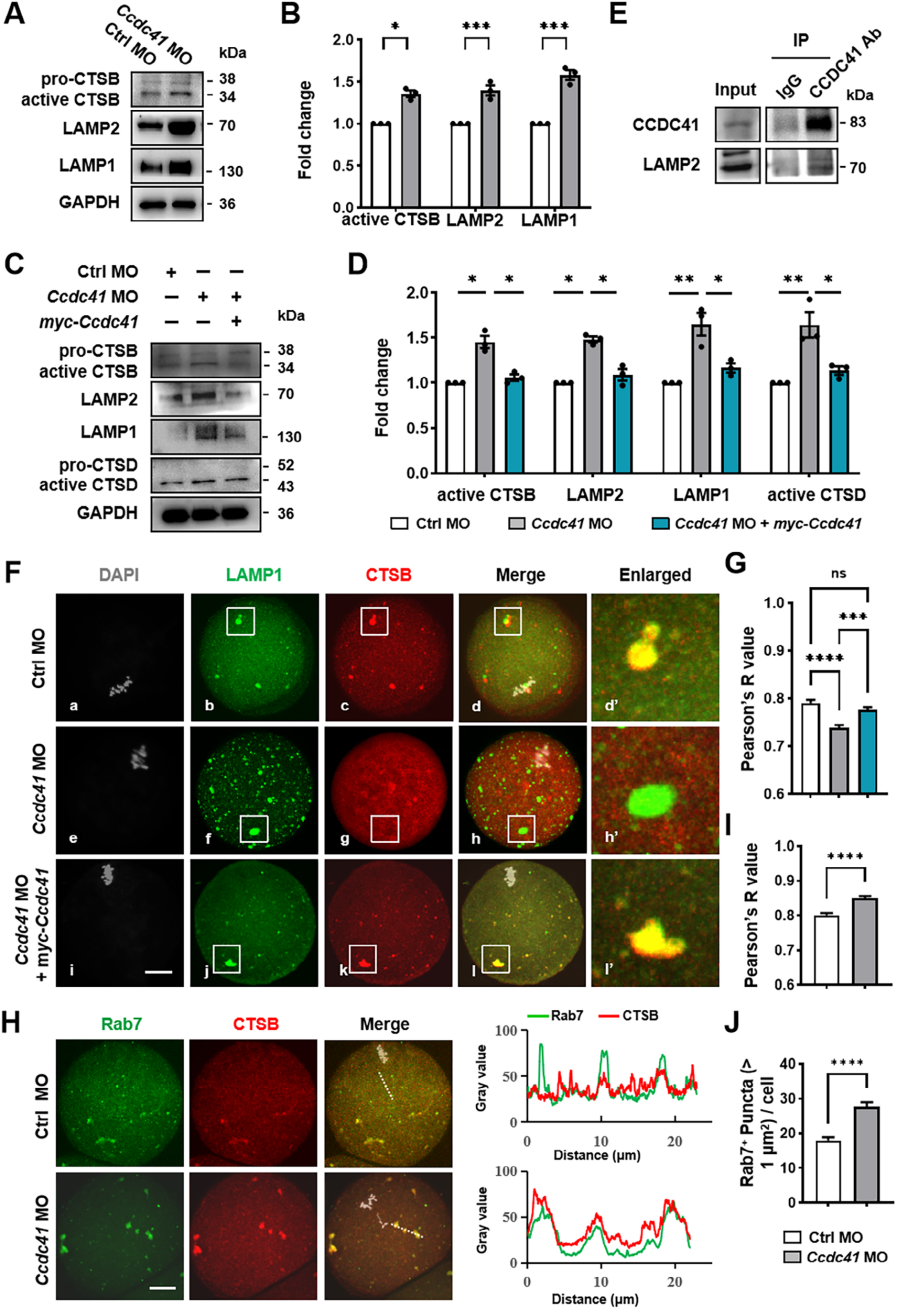
CCDC41 depletion disrupts cathepsin delivery to lysosomes. A) Immunoblot analysis of protein levels in Ctrl and *Ccdc41* MO oocytes. Each sample contained 180 oocytes. B) Quantitative analysis of active CTSB, LAMP2, and LAMP1 across Ctrl MO and *Ccdc41* MO groups. C) Immunoblot analysis of protein levels in Ctrl MO, *Ccdc41* MO, and *Ccdc41* MO + *myc‐Ccdc41* cRNA oocytes. Each sample contained 180 oocytes. D) Statistical evaluation of active CTSB, LAMP2, LAMP1, and active CTSD protein levels among Ctrl MO, *Ccdc41* MO, and *Ccdc41* MO + *myc‐Ccdc41* cRNA groups. E) Oocytes were immunoprecipitated with anti‐CCDC41 or IgG antibody conjugated to Protein‐A/G beads, and the precipitates were subjected to immunoblotting with anti‐CCDC41 and anti‐LAMP2. IP lysate contained 800 oocytes. F) Representative images showing the relationship between CTSB (red) and LAMP1 (green) in oocytes at 8 h of IVM. LAMP1 was visualized in green, CTSB in red, and DNA in gray. The white‐boxed area is magnified to reveal details. Scale bar = 20 µm. G) Statistical analysis of Pearson's correlation coefficients between CTSB and LAMP1 in Ctrl MO (n = 103), *Ccdc41* MO (n = 99), and *Ccdc41* MO + myc‐Ccdc41 cRNA (n = 99) groups. H) Representative images showing the relationship between CTSB (red) and Rab7 (green) in oocytes at 8 h of IVM. Rab7 was visualized in green LINE, CTSB in red, and DNA in gray. Scale bar = 20 µm. I) Statistical analysis of Pearson's correlation coefficients between CTSB and Rab7 in Ctrl MO (n = 105) and *Ccdc41* MO (n = 75) groups. J) Quantification of the number and area of RAB7 (>1 µm^2^) in Ctrl MO (n = 88) and *Ccdc41* MO (n = 61) oocytes. The experiment was performed three times independently. P‐values were calculated using one‐way ANOVA for D, G, or unpaired Student's t‐tests (two‐tailed) for B, I, and J. Statistical significance is indicated as ^*^P < 0.05, ^**^P < 0.01, ^***^P < 0.001, and ^****^P < 0.0001.

Consistent with lysosomal enlargement, LAMP1 and LAMP2 protein levels were significantly elevated in CCDC41‐depleted oocytes, an effect rescued by CCDC41 reconstitution (**Figure** **7**A,B). Co‐immunoprecipitation confirmed CCDC41 interaction with LAMP2 (Figure 7E), supporting its classification as a lysosome‐associated protein. Additionally, the active forms of cathepsin B and D (CTSB, CTSD) levels increased following CCDC41 knockdown and were restored by *myc‐Ccdc41* cRNA co‐injection (Figure 7C,D). In control oocytes, CTSB was localized to small, LAMP1‐positive cytoplasmic foci. Conversely, CCDC41 depletion caused CTSB to accumulate in enlarged, dispersed vesicles that exhibited significantly reduced co‐localization with LAMP1, a finding confirmed by Pearson's correlation analysis. This lysosomal delivery defect was rescued by the introduction of *myc‐Ccdc41* cRNA (Figure 7F,G). Given the role of late endosomes in delivering cathepsins to lysosomes,^[^ ^29^ ^]^ we investigated their morphology in CCDC41‐deficient oocytes. We observed that CCDC41 depletion significantly enlarged the area of late endosomes, labeled with Rab7.^[^ ^30^ ^]^ Intriguingly, this enlargement was accompanied by a marked increase in co‐localization with CTSB, as evidenced by a significantly elevated Pearson's coefficient (Figure 7H,I). Collectively, these data indicate that CCDC41 is essential for the proper lysosomal delivery of cysteine proteases, its deficiency disrupts this pathway, leading to the aberrant cytoplasmic accumulation of CTSB.

To assess CTSB activity and lysosomal acidity, we employed the Magic Red CTSB probe and the Lysosensor Green DND‐189 probe, respectively.^[^ ^31^, ^32^ ^]^ The specificity of both probes was confirmed by the observation that they produced only faint signals in oocytes injected with *Ctsb* MO (Figure S6E–G, Supporting Information). Live‐cell imaging then revealed a stark contrast between control and CCDC41‐depleted oocytes. While control oocytes exhibited a faint, diffuse Magic Red signal, CCDC41‐depleted oocytes displayed a significantly stronger and more widespread signal. This phenotype was effectively rescued by exogenous *Ccdc41* cRNA (Figure S6H,I, Supporting Information). A similar pattern was observed for Lysosensor Green: its fluorescence intensity was markedly elevated in CCDC41‐depleted oocytes, forming variable‐sized foci throughout the cytoplasm and accumulating around the spindle. In contrast, control oocytes showed only faint, localized signal near the chromosomes (Figure S6H,J, Supporting Information), and this alteration was also reversed by CCDC41 rescue (Figure S6H,J, Supporting Information). Collectively, these data demonstrate that CCDC41 depletion leads to the cytoplasmic hyperactivation of lysosomal proteases, likely due to their aberrant release from the enlarged late endosomes.

### 2.7 The Defected Meiotic Resumption and Anaphase Onset are Fundamentally Attributed to Mis‐Localized Cathepsins

**Figure 8:**
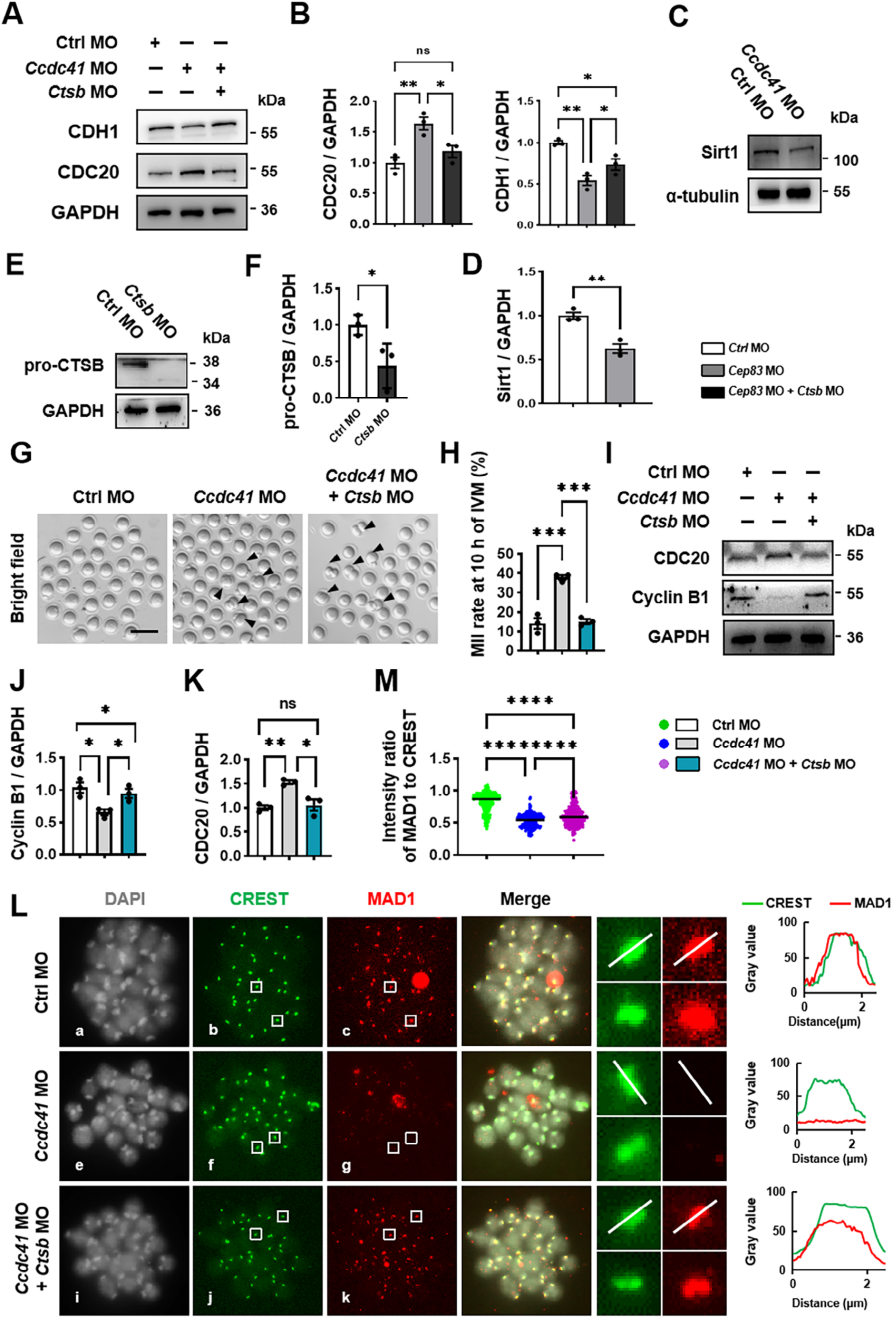
The defective meiotic resumption and anaphase onset are fundamentally attributed to mis‐localized cathepsins. A) Western blot images illustrating protein level alterations in GV oocytes treated with Ctrl MO, *Ccdc41*MO, and *Ccdc41* MO + *Ctsb* MO. B) Quantitative analysis of protein levels in Ctrl MO, *Ccdc41* MO, and *Ccdc41*MO + *Ctsb* MO‐treated GV oocytes, respectively. C) Western blot images illustrating Sirt1 alterations in GV oocytes treated with Ctrl MO and *Ccdc41* MO. D) Quantitative analysis of Sirt1 in Ctrl MO and *Ccdc41* MO‐treated GV oocytes, respectively. E) The protein level of pro‐CTSB were assessed by western blot in Ctrl MO and *Ctsb* MO oocytes. Each sample contained 180 oocytes. F) Quantitative assessment of pro‐CTSB protein level across different experimental groups of oocytes. G) Representative images of oocytes in Ctrl MO, *Ccdc41* MO, and *Ccdc41* MO + *Ctsb* MO groups at 8 h after GVBD (10 h of IVM). Arrows indicate enlarged PB1 in *Ccdc41* MO and *Ccdc41* MO + *Ctsb* MO groups. Scale bar = 200 µm. H) Statistical analysis of MetII oocytes at 8 h after GVBD in Ctrl MO (n = 100), *Ccdc41* MO (n = 100), and *Ccdc41* MO + *Ctsb* MO (n = 72) groups. I) Western blots of CDC20 and Cyclin B1 were detected by Western blot in Ctrl MO, *Ccdc41* MO, and *Ccdc41* MO + *Ctsb* MO oocytes. Each sample contained 90 oocytes. J‐K) Statistical comparison of CDC20 and Cyclin B1 protein levels in Ctrl MO, *Ccdc41* MO, and *Ccdc41* MO + *Ctsb* MO groups. L) SAC activity was assessed by the localization of MAD1 at the pro‐MetI stage in Ctrl MO, *Ccdc41* MO, and *Ccdc41* MO + *Ctsb* MO oocytes. Oocytes were fixed and immunostained for MAD1, CREST, and DNA at 6 h after GVBD. White lines indicate the direction of fluorescence intensity measurement. Fluorescence intensities of CREST (green line) and MAD1 (red line) are shown in the line graph. The distance is measured in µm. Scale bar = 2.5 µm. M) The relative fluorescence intensity of MAD1/CREST was measured in Ctrl MO (n = 301), *Ccdc41* MO (n = 313), and *Ccdc41* MO + *Ctsb* MO oocytes (n = 299). The experiment was performed three times independently. P‐values were calculated using one‐way ANOVA for B, J–M, or unpaired Student's t‐tests (two‐tailed) for D, F. Statistical significance is indicated as ^*^P < 0.05, ^**^P < 0.01, ^***^P < 0.001, and ^****^P < 0.0001.

Sirtuin 1 (Sirt1) is a well‐established stabilizer of the SAC component BubR1,^[^ ^7^ ^]^ which in turn stabilizes CDH1 during prophase.^[^ ^33^ ^]^ Critically, Sirt1 is a known substrate for CTSB,^[^ ^34^ ^]^ and CDC20 is a key substrate of the APC/C^CDH1^ complex.^[^ ^35^ ^]^ We therefore hypothesized that in CCDC41‐depleted oocytes, CTSB overactivation triggers Sirt1 degradation. This initiates a cascade: reduced CDH1 abundance leads to premature CDC20 activation, resulting in Cyclin B1 degradation and, ultimately, a delay in GVBD. To test this model, we found that Sirt1 protein levels were significantly reduced in CCDC41‐depleted oocytes at the GV stage (**Figure** **8**C,D). More importantly, co‐injection of *Ctsb* MO, which could efficiently knock down the protein level of CTSB (Figure 8E,F), rescued the CCDC41 depletion phenotype, restoring CDC20 and Cyclin B1 levels to near‐normal in *Ccdc41* MO‐treated oocytes (Figure 8A,B). Collectively, these data support a model where CCDC41 facilitates oocyte meiotic resumption by safeguarding the Sirt1‐BubR1‐CDH1/CDC20 pathway. This is accomplished through the regulation of cathepsin activity, which depends on maintaining their cytoplasmic homeostasis and efficient lysosomal delivery.

Following GVBD, a 5‐h incubation with Concanamycin A (ConA), a vacuolar H⁺‐ATPase inhibitor that blocks CTSB activation,^[^ ^16^ ^]^ significantly reduced abnormal MetI spindle formation and premature anaphase onset compared to DMSO controls (Figure S7A–C, Supporting Information). Consistently, ConA treatment increased Cyclin B1 levels while decreasing CDC20 and APC2 expression in CCDC41‐depleted oocytes at 6 h post‐GVBD (Figure S7D,E, Supporting Information). In addition, in CCDC41‐depleted oocytes, ConA treatment reduced the overall signal intensity of both GreenDND‐189 and Magic Red (Figure S8, Supporting Information). This effect may be attributed to the presence of V‐ATPases on various organelles, including endosomes and the Golgi apparatus, beyond just lysosomes.^[^ ^36^ ^]^ By blocking V‐ATPase activity, ConA might prevent the acidification of endosomes, including Rab7‐positive ones. This inhibition, in turn, reduces CTSB activity within these compartments, leading to the observed decrease in the overall Magic Red signal. However, our current data cannot distinguish whether CTSB is retained within endosomes or actively released into the cytosol, this distinction represents a crucial question for future investigation.

Aligning with ConA results, *Ctsb* MO reduced Magic Red and Lysosensor Green DND‐189 fluorescence intensity and distribution in MetI oocyte cytoplasm (Figure S9A–C, Supporting Information). *Ctsb* MO also reversed premature anaphase I onset and PB1 extrusion (Figure 8G,H), normalized CDC20 and Cyclin B1 levels (Figure 8I–K). Immunofluorescence of chromosome spreads revealed that CTSB knockdown restored MAD1 recruitment to centromeres in CCDC41‐depleted oocytes, with MAD1‐to‐CREST intensity ratios comparable to Ctrl MO oocytes (Figure 8L,M), indicating SAC recovery. Similarly, the CTSB inhibitor CA‐074 rescued CCDC41 knockdown‐induced phenotypes: reducing abnormal spindles and restoring MetII progression at 10 h (Figure S9D–F, Supporting Information), and increasing centromeric MAD1 signal intensity at 8 h (Figure S9G,H, Supporting Information). Western blot showed that CA‐074 prevented early Cyclin B1 degradation and CDC20 accumulation, though APC2 levels remained unchanged (Figure S9I,J, Supporting Information). Most metrics in the *Ccdc41* MO + CA‐074 group were comparable to Ctrl MO, except for APC2. Collectively, these findings demonstrate that CTSB hyperactivity drives SAC dysfunction and premature Cyclin B1 degradation in CCDC41‐deficient oocytes.

## 3 Discussion

This study reveals a new role of CCDC41 in regulating oocyte meiotic maturation in a manner of modulating vesicular transport and lysosomal function. The levels and regulatory mechanisms of Cyclin B1 are pivotal in governing the restart and orderly progression of meiosis in oocytes. APC/C^CDH1^ and APC/C^CDC20^ both degrade Cyclin B1, with APC/C^CDH1^ promoting G2 arrest and APC/C^CDC20^ advancing anaphase onset. APC/C^CDH1^ also targets CDC20 for degradation during G2.^[^ ^35^, ^37^ ^]^ Premature CDC20 activation disrupts Cyclin B1 accumulation, consequently delaying meiotic resumption in oocytes. Our findings indicate that CCDC41 stabilizes Sirt1 to maintain CDH1 levels, which in turn suppresses CDC20 activity. This precise regulation of CDH1 and CDC20 permits the accumulation of Cyclin B1 and promotes the timely resumption of meiosis in mouse oocytes. The SAC signaling delays anaphase until chromosomes are properly aligned, its inactivation enables APC/C^CDC20^ activation, degrading Cyclin B1 to initiate anaphase.^[^ ^38^ ^]^ Here, we found that CCDC41, a centromere‐associated protein, facilitates the recruitment of SAC proteins to the centromere and is essential for the establishment of SAC functionality in mouse oocytes. Depletion of CCDC41 resulted in premature SAC inactivation and APC/C^CDC20^ activation, thereby accelerating anaphase onset.

Evidence indicates a robust autophagic flux and lysosome‐dependent degradation persist during mitosis, and that finely controlled lysosomal leakage governs chromosome segregation.^[^ ^39^ ^]^ To date, there is a lack of definitive evidence linking lysosomal dysfunction to oocyte meiotic defects. In the process of cilia biogenesis, IFT20, a CCDC41 partner, induces autophagy through cilium‐based interactions with autophagy proteins and controls lysosome biogenesis by modulating the trafficking of acid hydrolase.^[^ ^40^, ^41^ ^]^ Our findings indicate that CCDC41 contributes to effective autophagic flux in mouse oocytes, at least in part by facilitating the delivery of cathepsins to lysosomes.

We found that the ablation of CCDC41 resulted in an increase in the average size of autophagic lysosomes and ELVA puncta, indicative of protein aggregate buildup attributable to defective lysosomal degradation,^[^ ^19^ ^]^ the lysosomal dysfunction was also implicated by lipid accumulation, a phenomenon accompanied by a compensatory increase in LAMP1 and LAMP2 expression. Lysosomal dysfunction is associated with the impaired delivery of hydrolytic enzymes, such as CTSB, to lysosomes. This impairment is linked to reduced fusion between late endosomes (LEs) and lysosomes.^[^ ^29^, ^30^ ^]^ This mis‐localization results in the widespread dispersion of CTSB throughout the cytoplasm, including its association with chromosomes and the spindle apparatus. Consequently, this not only compromised the degradation capacity within autolysosomes but also led to the premature degradation of essential proteins, such as those critical for the SAC function and spindle assembly, in non‐lysosomal compartments.^[^ ^17^ ^]^

Although CTSB activation is classically confined to lysosomes, our data from oocytes indicate the possibility of an alternative source of its activity outside of these organelles, a finding that warrants further investigation. The 33 kDa form of CTSB, generated in LEs and even the Golgi precursor, 46 kDa form, possesses significant degradative activity, indicating that active CTSB is not exclusively lysosomal.^[^ ^29^ ^]^ Our data suggest that CCDC41 is required for the efficient fusion of LEs with lysosomes. Its depletion is associated with a disruption of this process, which correlates with the formation of enlarged LEs and the appearance of active 33 kDa CTSB in the cytosol. We propose that this aberrant localization may set in motion a destructive feedback loop, whereby cytosolic CTSB could damage Golgi fragments and LE membranes, potentially promoting further CTSB release and exacerbating cellular dysfunction.^[^ ^29^ ^]^ Determining whether active CTSB is directly released from LEs, rather than lysosomes, in oocytes without CCDC41, will be a critical focus of our future research.

Previous studies have established a critical regulatory axis wherein Sirt1 maintains CDH1 activity during prophase by stabilizing BubR1, which in turn stabilizes CDH1 and thereby suppresses CDC20 activity.^[^ ^7^, ^33^ ^]^ Critically, our data reveal that hyperactivation of CTSB leads to Sirt1 degradation, disrupting this axis.^[^ ^34^ ^]^ Consequently, CDH1 levels are diminished, while CDC20 activity is inappropriately elevated, culminating in the degradation of Cyclin B1. This model elegantly explains the reduced Cyclin B1 levels and the delay in GVBD observed in CCDC41‐depleted oocytes. Collecting evidences suggest that cytoplasmic CTSB overactivation can trigger caspase activity by inducing mitochondrial damage and cytochrome C release. The resulting caspase activation leads to the degradation of the SAC proteins.^[^ ^17^, ^42^ ^]^ As we observed in CCDC41‐depleted oocytes, this SAC dysfunction, in turn, accelerates anaphase onset and causes chromosome separation errors. Consistently, treatment with Ca‐074, a mature CTSB inhibitor, or CTSB knockdown, rescued premature SAC inactivation and CDC20 accumulation, also reversed spindle abnormalities in CCDC41‐depleted oocytes.

CCDC201 and CCDC66, additional members of the CCDC protein family, are associated with oocyte development. A genome‐wide study revealed that CCDC201 is highly expressed in oocytes, and loss‐of‐function variants in this gene are linked to the pathogenesis of primary ovarian insufficiency. CCDC66 has been shown to localize to the meiotic spindle, where its knockdown compromises meiotic progression and chromosome segregation.^[^ ^43^, ^44^ ^]^ Despite recent advances, the precise roles and molecular underpinnings of CCDC201 and CCDC66 in oocyte biology remain largely elusive.

CCDC41 guides the trafficking of pre‐ciliary vesicles and the anchoring of the mother centriole to the apical membrane, a process that is also supported by Rab11a.^[^ ^26^ ^]^ Rab11a‐positive vesicles, by modulating F‐actin dynamics, drive spindle cortical migration during oocyte meiosis.^[^ ^45^, ^46^ ^]^ Similar to its role in cilium assembly, CCDC41 mediates the fusion of Rab11a‐positive vesicles with the plasma membrane, coupling the cytoplasmic F‐actin network to the cortical area, which is crucial for spindle migration to the cortex in mouse oocytes.

Taken together, this study provides evidence for a novel regulatory role for CCDC41 during oocyte meiotic maturation. Our data suggest that CCDC41 is a key contributor to proper meiotic progression, spindle assembly, and migration, potentially through its modulation of vesicular trafficking and lysosomal biogenesis. While CTSB activation is classically confined to lysosomes, our observations point to an alternative activity source in oocytes. This leads to the critical question of whether this activity stems from the direct release of CTSB from endosomes upon CCDC41 loss, a central hypothesis we aim to test in future studies.

## 4 Experimental Section

### Isolation, Oocyte and Somatic Cell Culture

All animal experiments were conducted in strict adherence to the policies and guidelines outlined in the Care and Use of Animals in Research and Teaching. The study was approved by the Animal Care and Use Committee of Capital Medical University under approval number AEEI‐2021‐215. Fully grown germinal vesicle (GV) oocytes were harvested from 3‐week‐old CB6F1 (Balb/c × C57BL/6) mice, which had been administered an intraperitoneal injection of 10 IU of pregnant mare serum gonadotropin (PMSG; Ningbo Second Hormone Factory, 110254564) to stimulate the development of preovulatory follicles. ≈44–48 h following PMSG injection, the mice were humanely euthanized using CO_2_. Subsequently, the ovaries were excised and transferred into minimum essential medium α (MEMα; Gibco, 12571‐063) supplemented with 3 mg/ml bovine serum albumin (BSA; Sigma–Aldrich, A1933), 10% fetal bovine serum (FBS; Gibco, 10099141), and 2.5 µm milrinone (Selleck, S2484). The ovaries were then punctured with a 27‐gauge needle to release the cumulus‐oocyte complexes (COCs). GV‐stage oocytes were carefully collected using mouth pipetting and maintained at the prophase arrest in MEMα containing milrinone, covered with paraffin oil (Sigma), and incubated at 37 °C in a humidified atmosphere with 5% CO_2_.

NIH‐3T3 cells were cultured in DMEM (Servicebio, G4511) containing 10% FBS (Gibco, 10099141) and 1% penicillin–streptomycin solution (Gibco, 15140122) at 37 °C in 5% CO2 atmosphere.

### Microinjection and Morpholino Oligo Interference

Fully grown GV oocytes were micromanipulated to receive injections of 5–10 pl of 1 mm morpholino (MO) antisense oligonucleotide targeting *Ccdc41* (5′‐GGCTGACATGCAATCAATAAGCTGT‐3′; Gene Tools) or Cathepsin B (5′‐GGATCAAGGACCACCACATCCTGCA‐3′; Gene Tools). A control MO oligonucleotide (5′‐CCTCTTACCTCAGTTACAATTTATA‐3′; Gene Tools) was used for injection in the control group. Following injection, the oocytes were maintained at the GV stage in M16 medium (Sigma–Aldrich, M7292) supplemented with 2.5 µm milrinone for 24 h to facilitate the degradation of the endogenous protein. Subsequently, the oocytes were transferred to fresh M16 medium devoid of milrinone for continued culture.

### Plasmid Construction and cRNA Production

The full‐length cDNA of wild‐type *Ccdc41* was subcloned into the pCS2‐myc or pcDNATM3.1(+)‐eGFP vectors (gifts from Prof. Zhenbo Wang from the Institute of Zoology, Chinese Academy of Sciences). The *Rab11a‐mCherry* plasmid was a generous gift from Melina Schuh.^[^ ^9^ ^]^ The *pCS2‐myc‐Ccdc41*, *mCherry‐Rab11a*, and *eGFP‐Ccdc41* plasmids were purified using the EndoFree Plasmid Midi Kit (CWBIO, CW2105S) and linearized at 37 °C. The linearized plasmids were then purified with the FastPure Gel DNA Extraction Mini Kit (Vazyme, DC301‐01). Complementary RNAs (cRNAs) were synthesized using the mMessage mMachine SP6 kit (ThermoFisher, AM1340) or the HisScribe T7 ARCA mRNA kit with tailing (New England Biolabs, E2050), following the manufacturer's protocols, and subsequently purified with the Monarch RNA cleanup kit (NEB, T2040L). Oocytes were injected with 10–15 pl of cRNA at a concentration of 1000–1500 ng µl^−1^ and then maintained at the GV stage for 8 h to allow for efficient protein synthesis. Following this period, the oocytes were released into milrinone‐free M16 medium for subsequent analysis.

The oocytes were prepared for live imaging using a Nikon NIS‐Elements Li2 microscope equipped with a Plan APO 60×/1.4 NA oil immersion objective. For Airyscan imaging, the samples were examined on a Zeiss Axio Imager A2 laser scanning microscope with a Plan APO 40×/0.95 NA objective, utilizing the Airyscan detection module.

### Antibodies

Rabbit anti‐CCDC41 antibody, mouse anti‐acetylated α‐tubulin antibody and rabbit anti‐GAPDH antibody were purchased from Sigma–Aldrich (HPA038161, T7451 and SAB4300645), rabbit anti–pCDK1Tyr15 antibody, rabbit anti‐ Cyclin B1 antibody and rabbit anti‐LC3 antibody were from Cell Signaling Technology (9111, 4138 and 4108), rabbit anti‐BUB3 antibody was from GeneTex (GTX113595), mouse anti‐CDC20 antibody, mouse anti‐CDH1 (Fzr1) antibody, mouse anti‐MAD1 antibody, mouse anti‐Ubiquitin antibody, mouse anti‐Rab11a antibody, mouse anti‐ATG5 antibody, mouse anti‐LAMP1 antibody and mouse anti‐Rab7 antibody were purchased from Santa Cruz Biotechnology (sc‐5296, sc‐56312, sc‐137025, sc‐8017, sc‐166523, sc‐133158, sc‐20011 and sc‐376362), rabbit anti‐γ‐tubulin antibody, rabbit anti‐eGFP antibody, rabbit anti‐CDK1 antibody, rabbit anti‐SQSTM1 antibody and Rabbit anti‐Cathepsin B antibody were purchased from Abcam (ab179503, ab184601, ab133327, ab56416 and ab214428), rabbit anti‐APC2 antibody, rabbit anti‐RAB11A antibody, rabbit anti‐RUFY1, mouse anti‐LAMP2 antibody and rabbit anti‐Cathepsin D antibody were purchased from Proteintech (13559‐1‐AP, 20229‐1‐AP, 13498‐1‐AP, 66301‐1‐1 g and 21327‐1‐AP), mouse anti‐GM130 antibody and mouse anti‐Pericentrin antibody were from BD Biosciences (610823 and 611814), rabbit anti‐Cathepsin B antibody was from ABclonal (A0967), mouse anti‐MYC antibody was from TransGen Biotechnology (HT101‐01), human anti‐Centromere protein antibody was from Antibodies (15‐234), rabbit anti‐LAMP1 antibody was from Developmental Studies Hybridoma Bank (Clone 1D4B), Alexa Fluor 488 goat anti‐rabbit IgG (H+L) was from Thermo Fisher Scientific (A‐11006), Alexa Fluor 488‐conjugated goat anti‐mouse IgG (H + L), Alexa Fluor 594‐conjugated goat anti‐rabbit IgG (H + L), HRP‐conjugated goat anti‐rabbit IgG (H + L) and HRP‐conjugated goat anti‐mouse IgG (H + L) were purchased from Zhongshan Golden Bridge Biotechnology (ZF‐0512, ZF‐0516, ZB‐2301 and ZB‐2305).

### Immunofluorescence and Microscopy

Oocytes were fixed in 4% paraformaldehyde (Sigma–Aldrich, 158127) in PEM buffer (100 mm PIPES, pH 6.9, 1 mm MgCl_2_, 1 mm EGTA) at room temperature for 30 min, followed by permeabilization in phosphate‐buffered saline (PBS; Sigma–Aldrich, P5493) supplemented with 0.5% Triton X‐100 (Sigma–Aldrich, T8787) for 10 min. After blocking with 10% normal goat serum (NGS; Zhongshan Golden Bridge Biotechnology, ZLI‐9 021) in PBS for 1 h, the oocytes were incubated with primary antibodies at 4 °C overnight. Subsequently, the oocytes were thoroughly washed in PBS containing 0.02% Triton X‐100 (PBST) and then incubated with the appropriate secondary antibodies for 1 h at room temperature. Following three 15‐min washes in PBST, the oocytes were mounted on slides using a mounting medium containing 6‐diamidino‐2‐phenylindole (DAPI; Vector Laboratories, H‐1 200). The samples were visualized using a laser‐scanning confocal microscope (Zeiss Axio Imager A2).

### Western Blot

Protein extracts from 50 to 200 oocytes were prepared using Laemmli Sample Buffer (BIO‐RAD, 1610737) supplemented with a Protease Inhibitor Cocktail (MCE, HY‐K0 016) and β‐mercaptoethanol (Sigma–Aldrich, M6250). The samples were heated at 100 °C for 5 min. Proteins were then separated by 10% SDS‐PAGE at 110 V for 1.5 h and transferred to a polyvinylidene fluoride (PVDF) membrane (Millipore, ISEQ00 010) at 250 mA for 2 h. The membrane was blocked in Tris‐buffered saline (TBS; containing 0.05% MgCl_2_, 150 mm NaCl, 10 mm Tris base, pH 7.4) with 0.1% Tween 20 (Solarbio, T82220) and 5% skimmed milk (Applygen, PS112L). The blocked membrane was incubated with the primary antibody at 4 °C overnight, followed by three 15‐min washes in TBST (TBS with 0.1% Tween 20). The membrane was then incubated with the secondary antibody for 1 h. After thorough washing, the blots were developed using an appropriate amount of ECL Plus substrate (Vazyme, E423). The relative intensity of the immunoblot signals was analyzed with Image Lab 3.0 software.

### Cell Transfection and Coimmunoprecipitation (Co‐IP) Assay

The stably transfected NIH 3T3 cells went through transient transfection with the *Myc* plasmid, using a DNA transfection reagent (Lipofectamine 3000; Invitrogen, L3000001) according to the manufacturer's instructions. At 48 h after transfection, cellular lysates were prepared by incubating the cells in lysis buffer (50 mm Tris–HCl [pH7.5], 150 mm NaCl, 1% Nonidet P‐40, 0.5% sodium deoxycholate) containing protease inhibitor cocktail for 30 min at 4 °C, then incubated with precoated Myc‐magnetic beads (LABLEAD, MNM‐25‐1000) for 1 h at 4 °C on a rotator. The Myc‐magnetic beads served as a negative control. The manufacturer's protocol was followed to wash the beads and elute the protein complex, which was subjected to Western blot to detect the Myc signal.

Oocytes were subjected to immunoprecipitation using the Pierce Crosslink Magnetic IP Co‐IP Kit (ThermoFisher, 88 805). Following the manufacturer's protocol, protein A&G agarose beads were pre‐cleared by washing twice with 1 × Modified Coupling Buffer. The beads were then incubated with the antibody for 15 min and subsequently washed three times with 1 × Modified Coupling Buffer. The antibodies or a non‐specific IgG control were crosslinked to the beads using DSS for 30 min. After crosslinking, the beads were washed three times with Elution Buffer and then twice with IP Lysis/Wash Buffer. Cellular lysates were prepared by incubating oocytes in IP Lysis/Wash Buffer supplemented with a protease inhibitor cocktail (MCE, HY‐K0010). The lysates were then incubated with the antibody‐crosslinked beads for 2 h at room temperature. Following incubation, the beads were washed twice with IP Lysis/Wash Buffer and once with ultrapure water. The immunoprecipitated antigens were eluted from the beads by resuspending them in Elution Buffer and boiling for 10 min. The eluted proteins were then analyzed by western blot.

### Chromosome Spreading

Oocytes were briefly treated with acidic Tyrode's solution (Sigma, T1788) to detach the zona pellucida. Following a short recovery period in MEMα medium, the oocytes were placed on glass slides and fixed in a 1% paraformaldehyde solution in distilled water, supplemented with 0.15% Triton X‐100 and 3 mm dithiothreitol. After air‐drying to prepare chromosome spreads, the slides were blocked with 10% NGS in PBS for 1 h at room temperature. The slides were then incubated with primary antibodies overnight at 4 °C. After three washes with PBS, the slides were incubated with secondary antibodies. The chromosomes were counterstained with DAPI, and the slides were imaged using a laser‐scanning confocal microscope (Zeiss Axio Imager A2).

### Drug treatment

All drugs were prepared as stock solutions in dimethylsulfoxide (DMSO) (Applichen, A3672). After proper treatment, oocytes were further incubated in fresh M16 with MG‐132 (Targetmol, T2154), Concanamycin A (MedChemExpress, HY‐N1724), Ca‐074 (MedChemExpress, HY‐103350), CP5V (MedChemExpress, HY‐130257), nocodazole (Sigma–Aldrich, M1404), Bafilomycin A1 (BafA1) (MedChemExpress, HY‐100558), and Brefeldin A (BFA) (MedChemExpress, HY‐16592) at final concentrations of 10 µm, 115 nm, 1 µm, 5 µm, 500 nm, 200 µM and 20 µM, respectively, for appropriate durations. Control groups received an equivalent volume of DMSO (at a final concentration of 1:1000 or less) for all drug treatments.

### Proximity Ligation Assay

The proximity ligation assay (PLA) was conducted using the Duolink In Situ Red Starter Kit for Mouse/Rabbit (Sigma–Aldrich, DUO92008). Oocytes were subjected to a series of fixation and permeabilization steps, akin to those used for immunofluorescence staining. The oocytes were then blocked with Duolink Blocking Buffer at 37 °C for 1 h, followed by incubation with the following diluted antibody pairs overnight at 4 °C: mouse anti‐Rab11a, mouse anti‐GM130 plus rabbit anti‐Rab11a, mouse anti‐GM130 plus rabbit anti‐CCDC41, and mouse anti‐Rab11a plus rabbit anti‐CCDC41. Subsequently, the oocytes were incubated with anti‐rabbit PLUS and anti‐mouse MINUS probes for 1 h, and then sequentially treated with 1 × ligase and 1 × polymerase for 30 and 100 min at 37 °C, respectively. Finally, the samples were mounted on slides using Duolink In Situ Mounting Medium containing DAPI for visualization.

### Fluorescent Detection of F‐Actin Assembly, CTSB Activity, and Lysosomal Acidit

To visualize F‐actin dynamics, oocytes were stained with 200 nM Cell Mask Green Actin Tracking Stain (Invitrogen, A57243) for 30 min in M2 medium at 37 °C. For assessing lysosomal pH, oocytes were incubated with 100 nM Lysosensor Green DND‐189 (YEASEN, 40767ES50) for 30 min. To quantify and monitor CTSB activity in live cells, oocytes were loaded with Magic Red Cathepsin B (diluted 1:250; Abcam, ab270772) for 30 min. Hoechst (Sigma–Aldrich, H33342) was included for nuclear staining alongside the aforementioned fluorescent probes. After labeling, the oocytes were subjected to six washes with fresh M2 medium and then placed in a 10 µl M2 medium drop, covered with mineral oil, in a 20‐mm glass‐bottom culture dish. Imaging was performed using a Nikon NIS‐Elements Li2 fluorescence microscope (Nikon, Japan), with the following excitation/emission settings: 365 nm/480 nm for Hoechst 33342, 488 nm/503–512 nm for green actin tracking, 540–560 nm/610 nm for Magic Red, and 443 nm/505 nm for Green DND‐189. Images from both control and experimental groups were captured under the same imaging conditions on the same microscope.

### Transmission electron microscopy (TEM)

Oocytes were fixed in 2.5% glutaraldehyde (Sigma–Aldrich, G5882) for a minimum of 2 days at 4 °C. Following fixation, they were rinsed three times for 10 min each in 0.1 m phosphate buffer (PB). A batch of 20–30 oocytes was then embedded in 1% agar (Sigma–Aldrich, A1 296) for 40 min. Subsequent dehydration was carried out through a graded series of ethanol concentrations, followed by infiltration with propylene oxide for solvent replacement. The samples were ultimately embedded in Epon 812 resin (Agar Scientific, R1045). Ultrathin sections, ranging from 60–80 nm in thickness, were cut using a diamond knife and mounted on copper grids. These sections were then contrasted with saturated uranyl acetate, followed by lead citrate. The prepared grids were examined and imaged using a FEI Tecnai 8482 Electron Microscope, which was operated at 120 kV.

### Quantification and Statistical Analysis

Data processing, statistical analysis, and graphical representation were conducted using Fiji (version 47), MATLAB (by Bitplane), Excel (© 2015 Microsoft), and GraphPad Prism (GraphPad Software, Inc.). In Fiji software, the “Analyze Particles” function was used to count LAMP1, RUFY1, LC3, Rab11a particles with an area greater than 1 µm^2^ and those with an area smaller than 1 µm^2^, respectively. Error bars in the graphs represent the mean ± standard error of the mean (SEM). The sample sizes and the specific statistical tests applied are detailed in the legends of the figures. All experimental procedures were repeated at least three times to ensure reliability of the results.

## Conflict of Interest

The authors declare no conflict of interest.

## Author Contributions

Y.T. and J.L. contributed equally to this work. W.M. and Y.T. conceived and designed the research; Y.T. and J.L. conducted the experiments; Y.T., J.L., J.W., Y.L. and W.M. analyzed the data. X.X., B.W., S.L., J.K., J.L., Y.Y., Y.Z. and Y.Z. provided materials, Y.T., J.L., and W.M. wrote the manuscript. All authors have agreed to submit this version for publication.

## Supporting information



Supporting Information

Supplemental Video 1

## Data Availability

The authors affirm that all data necessary to assess the conclusions presented in the paper are included within the paper and/or the accompanying Supplementary Materials. Should further data related to this study be required, interested parties may contact the authors to request additional information.
