# Supplementary material for: CCDC41 Drives Oocyte Meiotic Progression by Promoting Rab11a/Rab7‐Positive Vesicle Fusion with Target Membranes
Source: Adv Sci (Weinh). 2025 Dec 2;13(8):e04665. doi: 10.1002/advs.202504665 (PMC12884753; doi:10.1002/advs.202504665)
Supplement: Supplementary file 1 — Supporting Information [file ADVS-13-e04665-s002.docx]

Supporting Information

**CCDC41 Regulates Meiotic Resumption, Anaphase Onset and Asymmetric Division in Mouse Oocytes Through the Control of Lysosomal Biogenesis and Vesicle Trafficking Dynamics**

*Ying Tian, Jiatong Li, Jingyi Kang, Xiangning Xu, Bicheng Wang, Shuo Lou, Jingyu Li, Yuying Yang, Yanbing Zhang, Yangzi Zheng, Jing Weng, Yuanjing Liang, Wei Ma^*^*

**
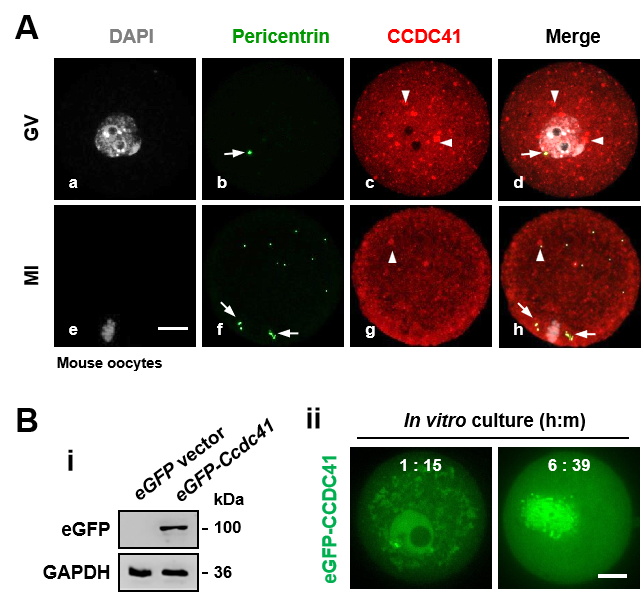
**

**Fig. S1**  **Subcellular localization of exogenous CCDC41 in oocytes.** A) Immunofluorescence imaging demonstrated the absence of overlap between CCDC41 (red) and Pericentrin (green), a component of acentriolar microtubule organizing centers (MTOCs), in mouse oocytes. Arrows indicate Pericentrin-positive signals, while arrowheads highlight the scattered punctate distribution of CCDC41, distinct from Pericentrin localization. Scale bar = 20 µm. B) Expression and subcellular localization of eGFP-CCDC41 in oocytes. (i) Western blot results confirmed the expression of eGFP-CCDC41 in oocytes, with each sample containing 60 oocytes. (ii) Live-cell confocal microscopy revealed the localization pattern of eGFP-CCDC41 in mouse oocytes. Scale bar = 20 µm.


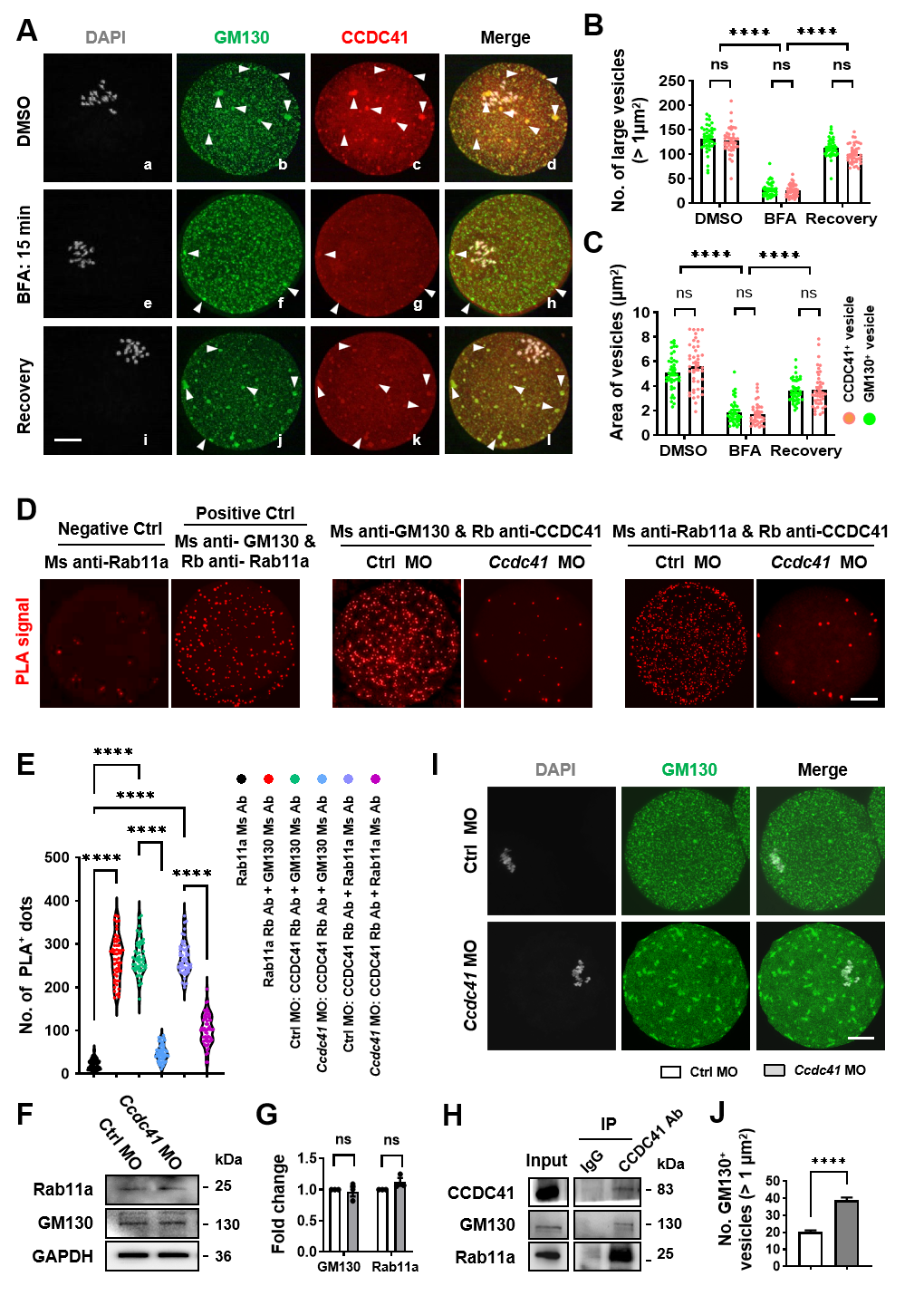


**Fig. S2** **CCDC41 is required for Rab11a dynamics in oocytes.** A) Representative images of MI oocytes. Scale bar = 20 µm. B) Statistical analysis of the number of CCDC41- or GM130-positive large vesicles (> 1 μm²) across DMSO, BFA-treated, and BFA-recovery groups. C) Statistical comparison of the mean area of CCDC41- or GM130-positive vesicles in DMSO, BFA-treated, and BFA-recovery groups. D) Representative images of proximity ligation assay (PLA) signals in Ctrl MO, *Ccdc41* MO, and *Ccdc41* MO + *myc-Ccdc41* cRNA groups, indicating the physical proximity of CCDC41 and GM130 or Rab11a. Scale bar = 20 µm. E) Quantitative analysis of PLA signal dots in each group. Dots with more than 100 pixels were included in the analysis. Sample sizes: Rab11a Ms ab group: n = 65; Rab11a Rb ab + GM130 Ms ab group: n = 57; Ctrl MO: CCDC41 Rb ab + GM130 Ms ab group: n = 54; *Ccdc41* MO: CCDC41 Rb ab + GM130 Ms ab group: n = 61; Ctrl MO: CCDC41 Rb ab + Rab11a Ms ab group: n = 53; *Ccdc41* MO: CCDC41 Rb ab + Rab11a Ms ab group: n = 62. F) Western blot analysis of Rab11a and GM130 expression in oocytes injected with *Ccdc41* MO or Ctrl MO. Each sample consisted of 80 oocytes. G) Quantitative assessment of protein levels. H) Co-immunoprecipitation (Co-IP) was conducted to validate the interaction between CCDC41 and GM130 and Rab11a. Lysates from 800 oocytes were incubated with anti-CCDC41 or IgG, and the IP eluates were probed with anti-CCDC41, anti-Rab11a, or anti-GM130 antibodies. I) Representative images of MI oocytes from the Ctrl MO, and *Ccdc41* MO groups. Scale bar = 20 µm. J) Statistical analysis of the number of large GM130^+^ vesicles (> 1 μm²) in Ctrl MO (n = 54) and *Ccdc41* MO (n = 50). The experiment was performed in triplicate. P-values were calculated using one-way ANOVA for B, C, E and unpaired Student’s t-tests (two-tailed) for G, J. Statistical significance is denoted as ****P < 0.0001, ns no significant.


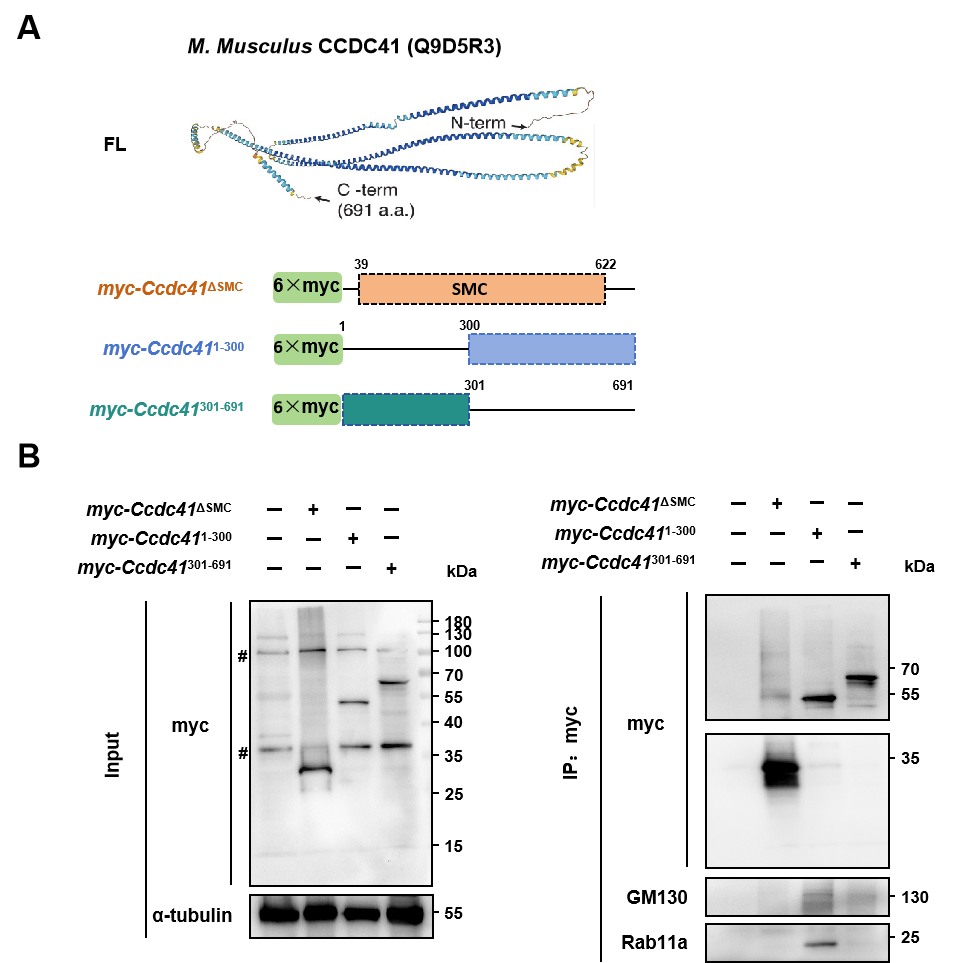


**Fig. S3** **Roles of the SMC domains in CCDC41.** A) Schematic representation of CCDC41 protein structure as predicted by AlphaFold. Deleted regions are denoted by dashed boxes. B) NIH 3T3 cells transfected with plasmids encoding the indicated proteins were cultured for 48 h, lysed, and subjected to immunoprecipitation using anti-myc magnetic beads. Input cell lysates and immunoprecipitates were analyzed by immunoblotting with antibodies against myc, α-tubulin, GM130, and Rab11a. The number sign indicates nonspecific bands.


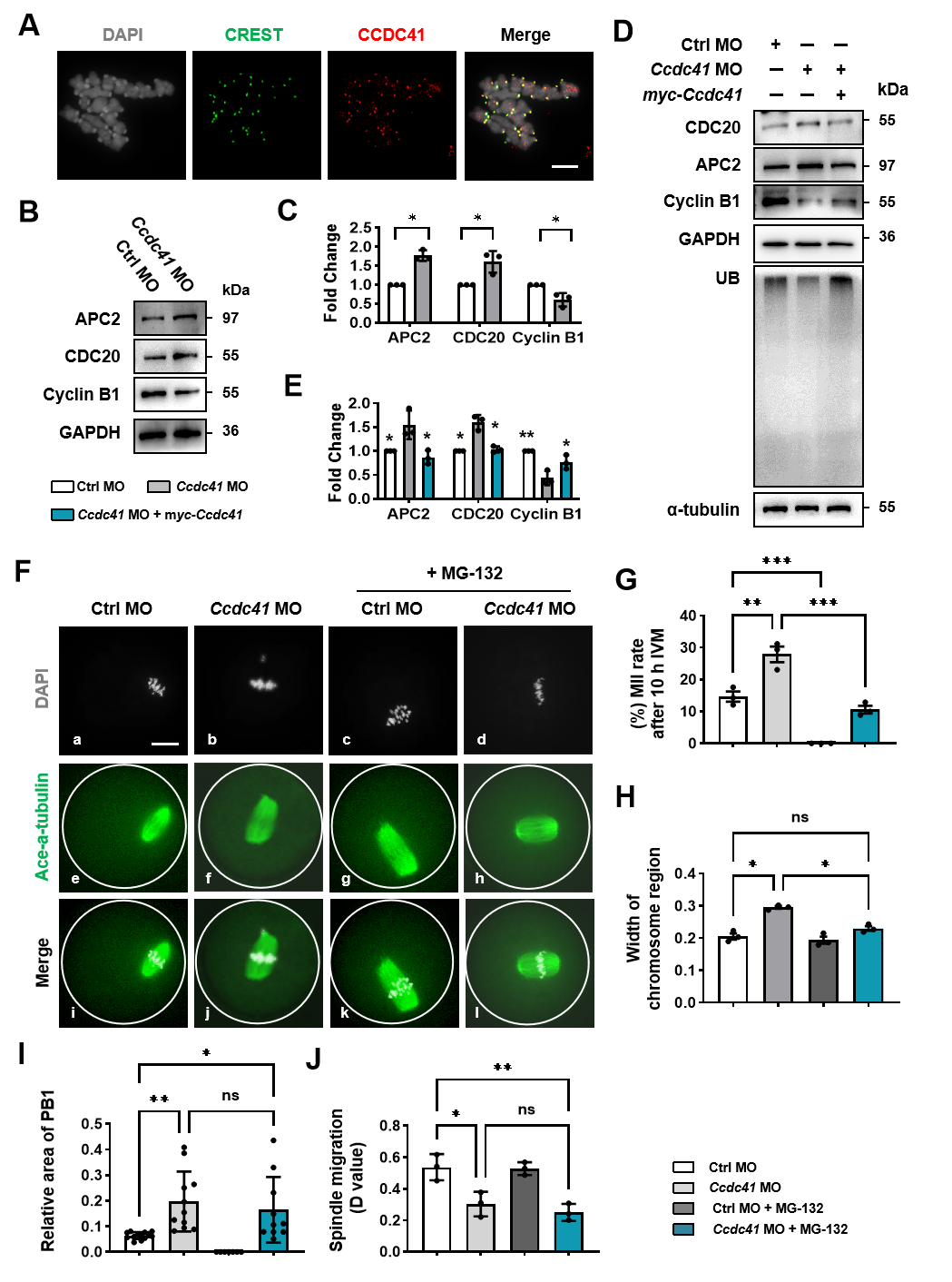


**Fig. S4 CCDC41 depletion triggers ubiquitination-mediated degradation of Cyclin B1.** A) Localization of CCDC41 (red) in MI oocytes, with kinetochores marked by CREST serum. Scale bar = 2.5 µm. B) Western blot analysis of protein expression in Ctrl and *Ccdc41* MO oocytes at 6 h post-GVBD. Western blots were probed with antibodies against Cyclin B1, CDC20, APC2, and GAPDH. Each sample consisted of 90 oocytes. C) Statistical analysis of protein levels across Ctrl MO and *Ccdc41* MO groups. D) Western blot analysis of protein levels in Ctrl MO, *Ccdc41* MO, and *Ccdc41* MO + *myc-Ccdc41* cRNA oocytes at 6 h post-GVBD. The blots were incubated with antibodies specific for Cyclin B1, CDC20, APC2, Ubiquitin (UB), and GAPDH. Each sample contained 90 oocytes. E) Statistical analysis of protein levels. F) Representative images of MI oocytes. Scale bar = 20 µm. G-J) Statistical analysis of the MII rate at 10 h (%) (G), width of chromosome region (H) the relative size of PB1 (I), and the D value of spindle migration (J) in Ctrl MO (n = 91), *Ccdc41* MO (n = 132), Ctrl MO + MG132 (n = 108), and *Ccdc41* MO + MG132 (n = 111) groups. The experiment was conducted in triplicate. P-values were calculated using unpaired Student’s t-tests (two-tailed) or one-way ANOVA. Statistical significance is indicated as *P < 0.05, **P < 0.01, ***P < 0.001.


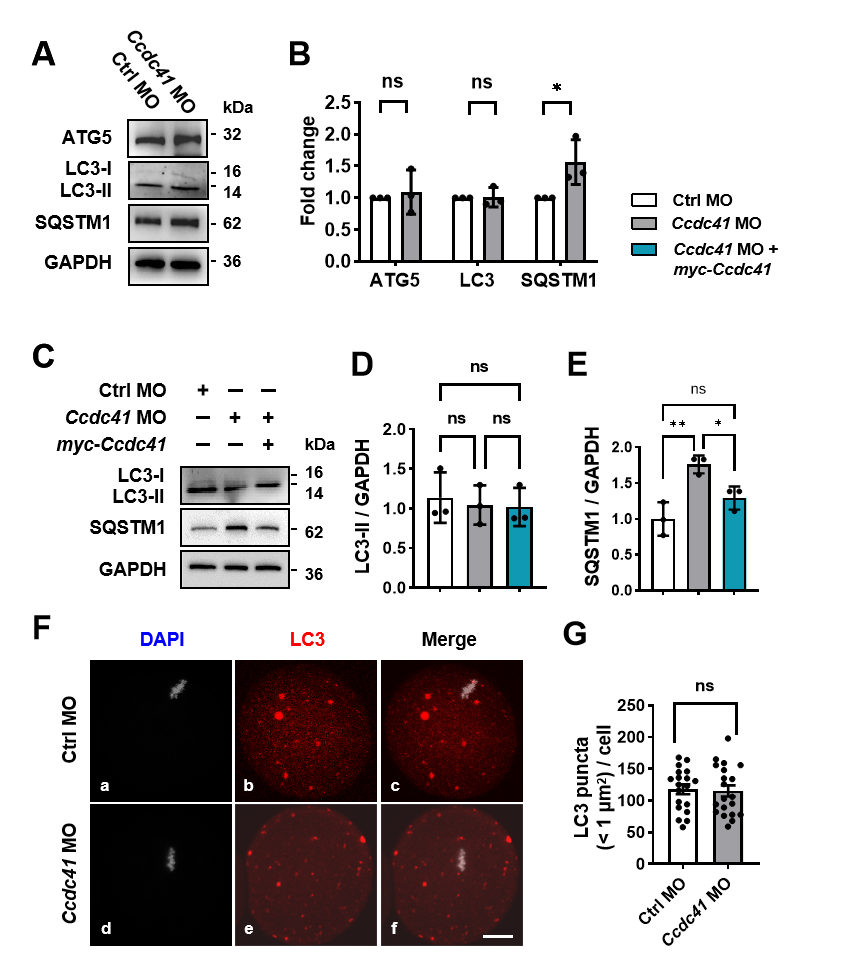


**Fig. S5**  **CCDC41 knockdown inhibited autophagy in oocytes.** A) Immunoblot analysis of autophagy markers LC3, SQSTM1, and ATG5 in Ctrl and *Ccdc41* MO oocytes. Each sample contained 60 oocytes. B) Statistical analysis of LC3, SQSTM1, and ATG5 protein levels across Ctrl MO and *Ccdc41* MO groups. C) Immunoblot analysis of LC3 and SQSTM1 in Ctrl MO, *Ccdc41* MO, and *Ccdc41* MO + *myc-Ccdc41* cRNA oocytes. Each sample contained 60 oocytes. D-E) Quantitative analysis of LC3 and SQSTM1 protein levels. F) Immunofluorescence images of oocytes with or without CCDC41 depletion by MO, immunolabeled with anti-LC3 antibody. Scale bar = 20 µm. G) Quantification of LC3 dot (< 1 μm^2^) numbers following *Ccdc41* MO treatment. Ctrl MO: n = 19; *Ccdc41* MO: n = 20. The experiment was repeated three times independently. P-values were calculated using one-way ANOVA for D, E or unpaired Student’s t-tests (two-tailed) for B, G. Statistical significance is indicated as *P < 0.05, **P < 0.01

**
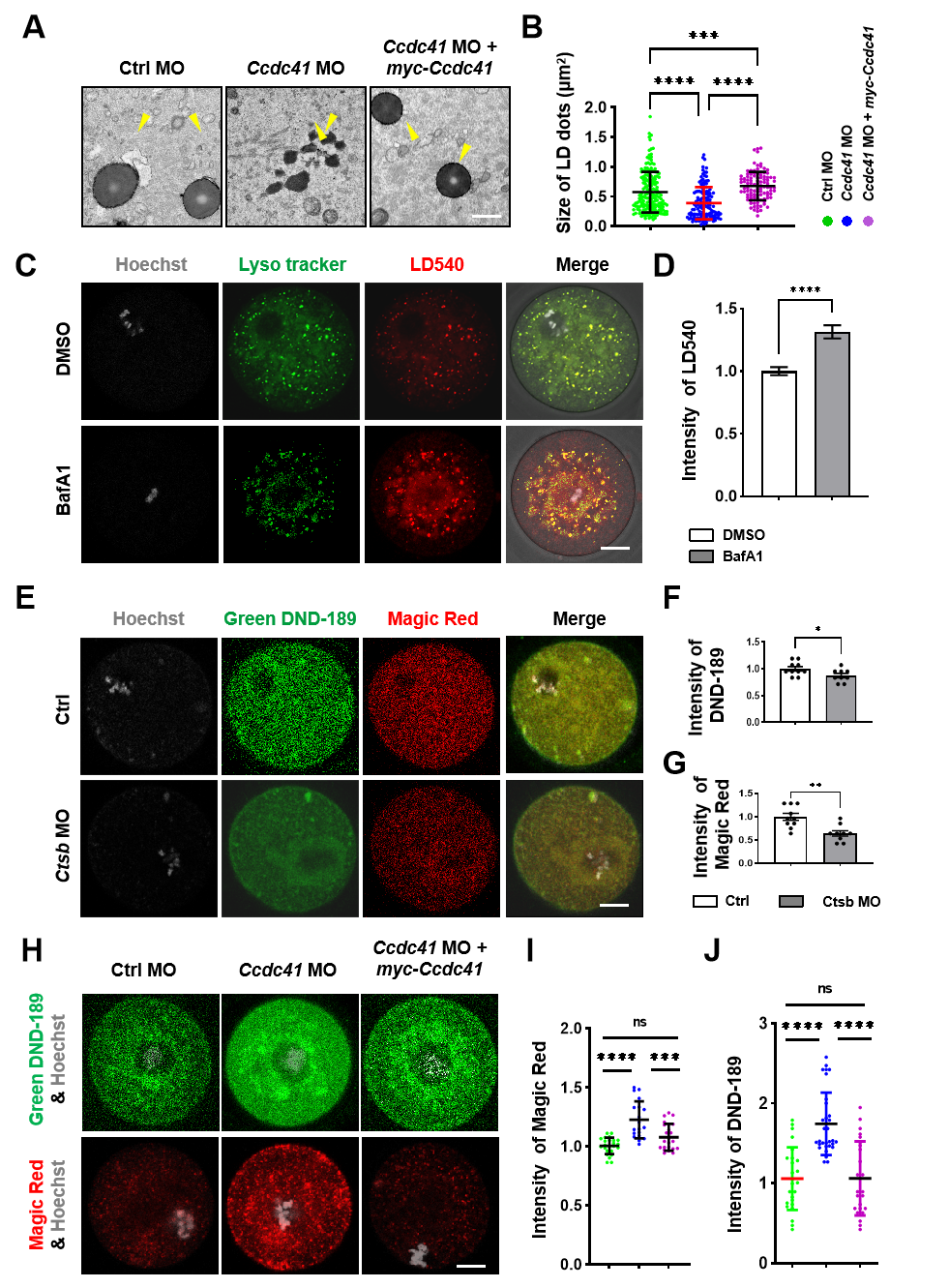
Fig. S6 CCDC41 is required for lipid droplet breakdown.** A) Transmission electron microscopy images of Ctrl MO, *Ccdc41* MO, and *Ccdc41* MO + *myc-Ccdc41* cRNA oocytes at the MI stage. Arrows indicate lipid droplets (LD). B) The graph shows the quantification of the average size of LD dots. Ctrl MO group: n = 180; *Ccdc41* MO group: n = 138; *Ccdc41* MO + *myc-Ccdc41* cRNA group: n = 104. C) Representative confocal images of oocytes labeled with the lysosome marker-Lysotracker and lipid droplet marker-LD540. Maximal Z projections across the equatorial region of each oocyte. Scale bar = 20 μm. D) Statistical analysis of LD540 fluorescence intensity in Ctrl (n = 35) and Baf A1 (n = 39) oocytes. E) Confocal images of MI oocytes stained with LysoSensor Green DND-189 and CTSB activity probe Magic Red. Maximal Z projections across the equatorial region of each oocyte. Scale bar = 20 μm. F-G) Statistical analysis of DND-189 and Magic Red fluorescence intensity in Ctrl (n = 10) and *Ctsb* MO (n = 9) oocytes. H) Representative Confocal images of oocytes labeled with LysoSensor Green DND-189 and Magic Red. Maximal Z projections across the equatorial region of each oocyte. Scale bar = 20 μm. I) Quantification of the mean Magic Red intensity in Ctrl MO (n = 31), *Ccdc41* MO (n = 27), and *Ccdc41* MO + *myc-Ccdc41* cRNA (n = 22) oocytes. J) Statistical analysis of DND-189 fluorescence intensity in Ctrl MO (n = 25), *Ccdc41* MO (n = 32), and *Ccdc41* MO + *myc-Ccdc41* cRNA (n = 24) oocytes. The experiment was performed three times independently. P-values were calculated using one-way ANOVA or unpaired Student’s t-tests (two-tailed) for D. Statistical significance is indicated as *P < 0.05, **P < 0.01, ***P < 0.001 and ****P < 0.0001


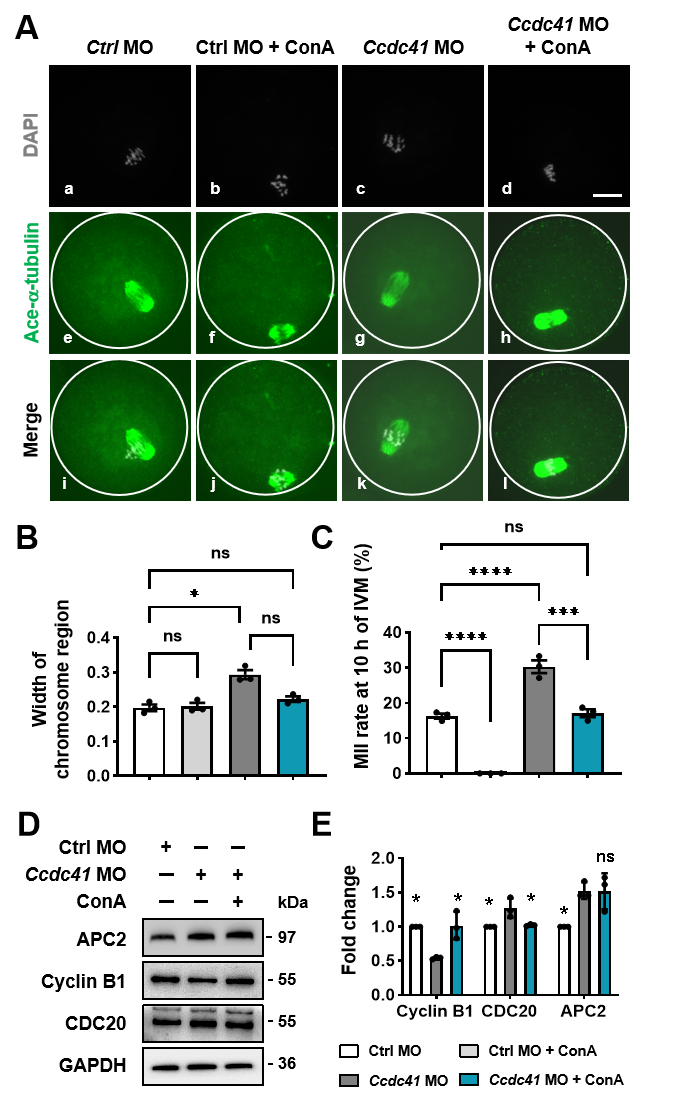


**Fig. S7**  **ConA restores spindle morphology and the initiation of anaphase I in CCDC41-depleted oocytes.** A) Representative images of oocytes immunolabeled with anti-acetylated α-tubulin (Ace-α-tubulin) in Ctrl MO, *Ccdc41* MO, Ctrl MO + ConA, and *Ccdc41* MO + ConA groups at 6 h after GVBD. Scale bar = 20 µm. B) Quantification of width of chromosome region in Ctrl MO (n = 96), *Ccdc41* MO (n = 102), Ctrl MO + ConA (n = 98), and *Ccdc41* MO + ConA (n = 97) groups. C) Quantification of the MII rate at 10 h (%) in Ctrl MO (n = 82), *Ccdc41* MO (n = 78), Ctrl MO + ConA (n = 88), and *Ccdc41* MO + ConA (n = 93) groups. D) Immunoblot images of APC2, Cyclin B1, and CDC20 in Ctrl MO, *Ccdc41* MO, and *Ccdc41* MO + ConA oocytes. Each sample contained 90 oocytes. E) Statistical comparison of APC2, Cyclin B1, and CDC20 protein levels in Ctrl MO, *Ccdc41* MO, and *Ccdc41* MO + ConA groups. The experiment was performed three times independently. P-values were calculated using one-way ANOVA. Statistical significance is indicated as *P < 0.05, ***P < 0.001, and ****P < 0.0001.

**Fig. S8**  **V-ATPase inhibition with ConA inhibits intracellular acidification and CTSB activation in CCDC41-depleted oocytes.** A). Confocal images of GV oocytes stained with LysoSensor Green DND-189 and CTSB activity probe Magic Red. Maximal Z projections across the equatorial region of each oocyte. Scale bar = 20 μm. B). Statistical analysis of DND-189 and Magic Red fluorescence intensity in Ctrl (n = 29), *Ccdc41* MO (n = 28) and *Ccdc41* MO + ConA (n = 27) oocytes. The experiment was performed three times independently. P-values were calculated using one-way ANOVA. Statistical significance is indicated as **P < 0.01 and ****P < 0.0001.


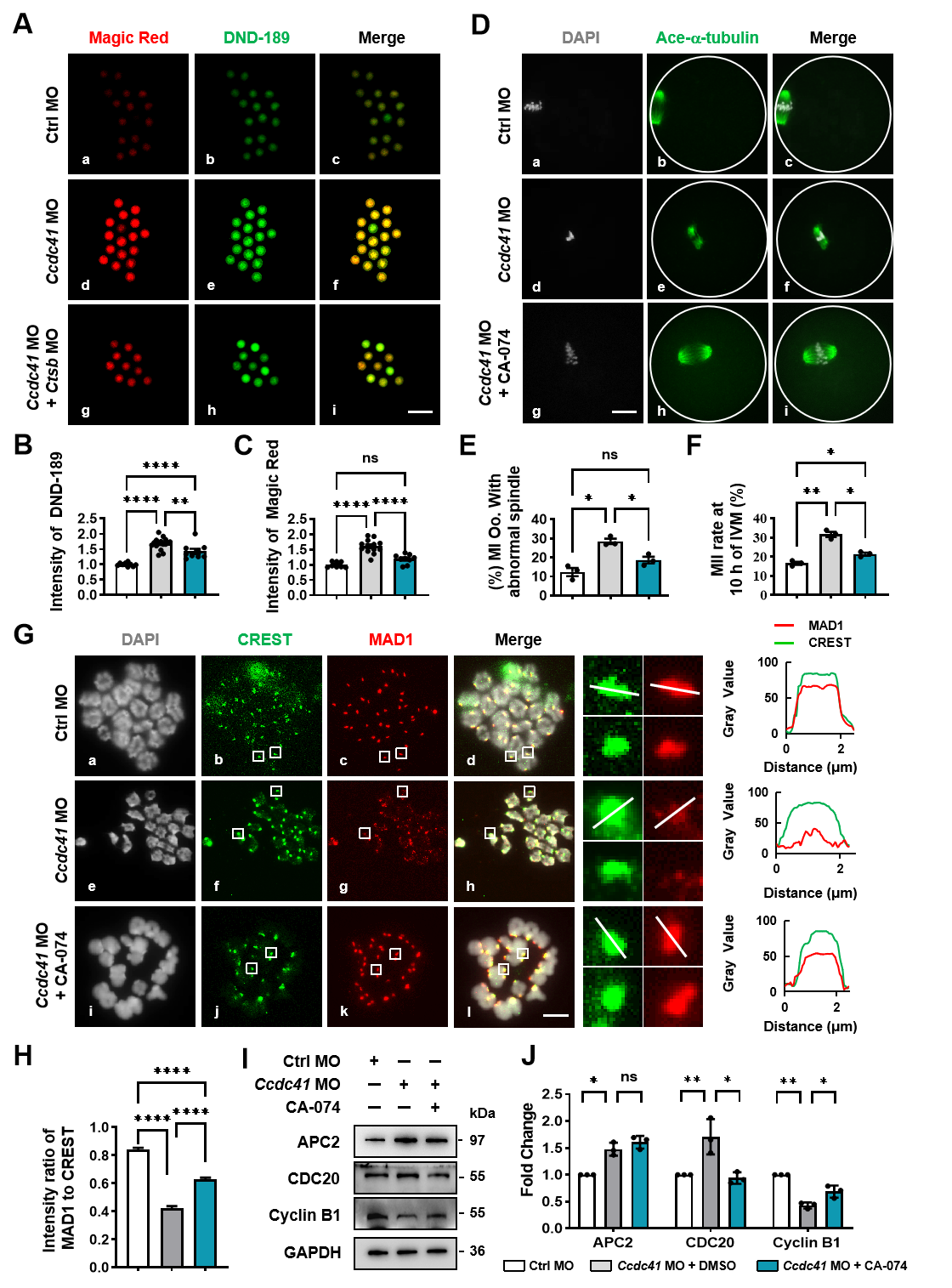


**Fig. S9**  **CTSB inhibition restores spindle morphology and SAC function in CCDC41-depleted oocytes.** A) Confocal images of MI oocytes stained with the CTSB activity probe Magic Red and Lysosensor Green DND-189. Scale bar = 200 μm. B-C) Quantification of the mean Lysosensor Green DND-189 and Magic Red intensity from the experiment showed in A. Ctrl MO group: n = 9; *Ccdc41* MO group: n = 16; Ctrl MO + *Ctsb* MO group: n = 9. A) Representative images of oocytes immunolabeled with anti-ace-α-tubulin (green) in Ctrl MO, *Ccdc41* MO, and *Ccdc41* MO + CA-074 groups at 6 h post-GVBD. DNA was labeled in gray with DAPI. Scale bar = 20 µm. B) Quantification of the MII rate at 10 h (%) in Ctrl MO (n = 75), *Ccdc41* MO (n = 63), and *Ccdc41* MO + CA-074 (n = 68) groups. C) Quantification of the percentage of abnormal spindles in Ctrl MO (n = 86), *Ccdc41* MO (n = 91), and *Ccdc41* MO + CA-074 (n = 92) groups. D) The relative fluorescence intensity of MAD1/CREST was measured in Ctrl MO (n = 315), *Ccdc41* MO (n = 272), and *Ccdc41* MO + CA-074 oocytes (n = 299). E) SAC activity was assessed by the localization of MAD1 on centromeres at the prometaphase I stage in Ctrl MO, *Ccdc41* MO, and *Ccdc41* MO + CA-074 oocytes. Oocytes were fixed and immunostained for MAD1, CREST, and DNA at 6 h post-GVBD. Scale bar = 2.5 µm. White lines indicate the direction for measuring fluorescence intensity. Fluorescence intensities of CREST (green line) and MAD1 (red line) are showed in the line graph. The distance is measured in μm. F) The protein levels of APC2, CDC20 and Cyclin B1 were detected by western blot in Ctrl MO, *Ccdc41* MO, and *Ccdc41* MO + CA-074 oocytes. Each sample contained 90 oocytes at 4 h post-GVBD. G-I) Statistical comparison of APC2, CDC20 and Cyclin B1 protein levels. The experiment was performed three times independently. p-values were calculated using one-way ANOVA. Statistical significance is indicated as *P < 0.05, **P < 0.01, and ***P < 0.001.

**Video S1** The oocytes were injected with mCherry-tagged Rab11a vector.

Representative videos showing the outward transport of vesicles during continuous *in vitro* culture at 6 - 8 h among the Ctrl MO (A), *Ccdc41* MO (B) and *Ccdc41* MO + *myc-Ccdc41* cRNA (C) groups. Scale bar = 20 µm.
